# An immunodominant NP_105–113_-B*07:02 cytotoxic T cell response controls viral replication and is associated with less severe COVID-19 disease

**DOI:** 10.1038/s41590-021-01084-z

**Published:** 2021-12-01

**Authors:** Yanchun Peng, Suet Ling Felce, Danning Dong, Frank Penkava, Alexander J. Mentzer, Xuan Yao, Guihai Liu, Zixi Yin, Ji-Li Chen, Yongxu Lu, Dannielle Wellington, Peter A. C. Wing, Delaney C. C. Dominey-Foy, Chen Jin, Wenbo Wang, Megat Abd Hamid, Ricardo A. Fernandes, Beibei Wang, Anastasia Fries, Xiaodong Zhuang, Neil Ashley, Timothy Rostron, Craig Waugh, Paul Sopp, Philip Hublitz, Ryan Beveridge, Tiong Kit Tan, Christina Dold, Andrew J. Kwok, Charlotte Rich-Griffin, Wanwisa Dejnirattisa, Chang Liu, Prathiba Kurupati, Isar Nassiri, Robert A. Watson, Orion Tong, Chelsea A. Taylor, Piyush Kumar Sharma, Bo Sun, Fabiola Curion, Santiago Revale, Lucy C. Garner, Kathrin Jansen, Ricardo C. Ferreira, Moustafa Attar, Jeremy W. Fry, Rebecca A. Russell, COMBAT Consortium, Hans J. Stauss, William James, Alain Townsend, Ling-Pei Ho, Paul Klenerman, Juthathip Mongkolsapaya, Gavin R. Screaton, Calliope Dendrou, Stephen N. Sansom, Rachael Bashford-Rogers, Benny Chain, Geoffrey L. Smith, Jane A. McKeating, Benjamin P. Fairfax, Paul Bowness, Andrew J. McMichael, Graham Ogg, Julian C. Knight, Tao Dong

**Affiliations:** 1 MRC Human Immunology Unit, MRC Weatherall Institute of Molecular Medicine, Radcliffe Department of Medicine, University of Oxford, Oxford, UK; 2 Chinese Academy of Medical Science Oxford Institute, University of Oxford, Oxford, UK; 3 Wellcome Centre for Human Genetics, University of Oxford, Oxford, UK; 4 Nuffield Department of Medicine, University of Oxford, Oxford, UK; 5 CAMS Key Laboratory of Tumor Immunology and Radiation Therapy, Xinjiang Tumor Hospital, Xinjiang Medical University, Urumqi, China; 6 Nuffield Department of Orthopaedics, Rheumatology and Musculoskeletal Sciences, University of Oxford, Oxford, UK; 7 Beijing You’an Hospital, Capital Medical University, Beijing, China; 8 Department of Pathology, University of Cambridge, Cambridge, UK; 9 Single Cell Facility, MRC Weatherall Institute of Molecular Medicine, University of Oxford, Oxford, UK; 10 Sequencing Facility, MRC Weatherall Institute of Molecular Medicine, University of Oxford, Oxford, UK; 11 Flow Cytometry Facility, MRC Weatherall Institute of Molecular Medicine, University of Oxford, Oxford, UK; 12 Genome Engineering Facility, MRC Weatherall Institute of Molecular Medicine, University of Oxford, Oxford, UK; 13 Virus Screening Facility, MRC Weatherall Institute of Molecular Medicine, University of Oxford, Oxford, UK; 14 Oxford Vaccine Group, Department of Paediatrics, and NIHR Oxford Biomedical Research Centre, Centre for Clinical Vaccinology and Tropical Medicine, University of Oxford, Oxford, UK; 15 Department of Oncology, University of Oxford, Oxford, UK; 16 MRC Weatherall Institute of Molecular Medicine, University of Oxford, Oxford, UK; 17 Helmholtz Center Munich—German Research Center for Environmental Health, Institute of Computational Biology, Neuherberg, Germany; 18 Translational Gastroenterology Unit, Nuffield Department of Medicine, University of Oxford, Oxford, UK; 19 Kennedy Institute for Rheumatology, University of Oxford, Oxford, UK; 20 ProImmune Limited, Oxford, UK; 21 Sir William Dunn School of Pathology, University of Oxford, Oxford, UK; 22 Institute of Immunity and Transplantation, University College London, London, UK; 23 James & Lillian Martin Centre, Sir William Dunn School of Pathology, University of Oxford, Oxford, UK; 24 Peter Medawar Building for Pathogen Research, University of Oxford, Oxford, UK; 25 Dengue Hemorrhagic Fever Research Unit, Office for Research and Development, Faculty of Medicine, Siriaj Hospital, Mahidol Unviversity, Bangkok, Thailand; 26 Division of Infection and Immunity, University College London, London, UK

**Keywords:** Immunological memory, Viral infection

## Abstract

NP_105–113_-B*07:02-specific CD8^+^ T cell responses are considered among the most dominant in SARS-CoV-2-infected individuals. We found strong association of this response with mild disease. Analysis of NP_105–113_-B*07:02-specific T cell clones and single-cell sequencing were performed concurrently, with functional avidity and antiviral efficacy assessed using an in vitro SARS-CoV-2 infection system, and were correlated with T cell receptor usage, transcriptome signature and disease severity (acute *n* = 77, convalescent *n* = 52). We demonstrated a beneficial association of NP_105–113_-B*07:02-specific T cells in COVID-19 disease progression, linked with expansion of T cell precursors, high functional avidity and antiviral effector function. Broad immune memory pools were narrowed postinfection but NP_105–113_-B*07:02-specific T cells were maintained 6 months after infection with preserved antiviral efficacy to the SARS-CoV-2 Victoria strain, as well as Alpha, Beta, Gamma and Delta variants. Our data show that NP_105–113_-B*07:02-specific T cell responses associate with mild disease and high antiviral efficacy, pointing to inclusion for future vaccine design.

## Abstract (web summary)

Peng et al. find that immunodominant cytotoxic T lymphocytes (CTLs) specific for NP_105–113_-B*07:02 are associated with reduced COVID-19 severity. Mechanistically, NP_105–113_-B*07:02-specific CTLs show potent antiviral functionality and may represent rational T cell vaccine targets.

## Main

CD8^+^ T cells play a well-documented role in clearing viral infections. Immunodominance is a central feature of CD8^+^ T cell responses in viral infections and understanding the nature of this response for a given infection, where they are shown to be protective, will be critical for the design of vaccines aiming to elicit optimal CD8^+^ T cell responses^1,2^.

The role of the immunodominant cytotoxic T cell immune response in protection and potential disease pathogenesis of SARS-CoV-2 infection is currently poorly defined. We and others have identified immunodominant T cell epitopes restricted by common human leukocyte antigen (HLA) types^3–6^; in particular, we found multiple dominant epitopes in nucleoprotein (NP) restricted by HLA-B*07:02, -B*27:05, -B*40:01, -A*03:01 and -A*11:01. We also found that multi-functional NP and membrane (M) CD8^+^ T cell responses are associated with mild disease; NP is one of the most common targets for CD8^+^-dominant T cell responses in SARS-CoV-2 infection^3^.

Among the dominant epitopes identified to date, NP_105–113_-B*07:02 appears to be among the most dominant^3,4,6,7^; notably, no variants are found within this epitope from over 300,000 sequences in COG-UK global sequence data alignment^8^. This suggests that this epitope would be a good target for inclusion within an improved vaccine design, expanded to stimulate effective CD8^+^ T cell responses, as well as neutralizing antibodies, to protect against newly emergent viral strains that escape antibody responses to spike in some cases^9^.

Biased *TRBV27* gene usage, with long CDR3β loops preferentially expressed in NP_105–113_-B*07:02-specific T cell receptor (TCR), has been observed in both unexposed and COVID-19-recovered individuals^10^. The present study suggested a role for cross-reactive responses in COVID-19 based on pre-existing immunity to seasonal coronaviruses or other pathogens. However, a subsequent study suggested that the immunodominant NP_105–113_-B*07:02 CD8^+^ T cell responses are unlikely to arise from pre-existing cross-reactive memory pools, but rather represent a high frequency of naive T cell precursors found across HLA-B*07:02-expressing individuals^7^.

In this study, we present﻿ an in-depth analysis to explore correlations across NP_105–113_-B*07:02-specific T cell responses, TCR repertoires and disease severity. We saw stronger overall T cell responses in individuals recovered from severe COVID, which may be explained by high exposure to viral protein; however, we found an immunodominant epitope response (HLA-B*07:02 NP_105–113_-specific CD8^+^) which significantly associated with mild cases. Importantly, this epitope is one of the most dominant CD8^+^ T cell epitopes reported so far by us and others. We examined potential mechanisms of protection using single-cell transcriptome analysis, and functional evaluation of expanded T cell clones bearing the same TCRs as those identified in single-cell analysis. We also assessed the ability of T cell lines and clones to mount effective effector function against cells infected with live SARS-CoV-2 virus and vaccinia virus-expressing SARS-CoV-2 proteins. We found that NP_105–113_-B*07:02 is the dominant NP response in HLA-B*07:02-positive patients with mild symptoms, with high frequency and higher magnitude when compared with severe cases. Single-cell analysis revealed that preserved beneficial functional phenotypes are associated with protection from severe illness and have better overall antiviral function. In addition, NP_105–113_-B*07:02-specific T cells can recognize the naturally processed epitope in live virus and recombinant vaccinia virus-infected cells, which correlates with antiviral efficacy.

## Results

### NP_105–113_-B*07:02-specific T cell responses are stronger in patients recovered from mild COVID-19 infection

**Fig. 1:**
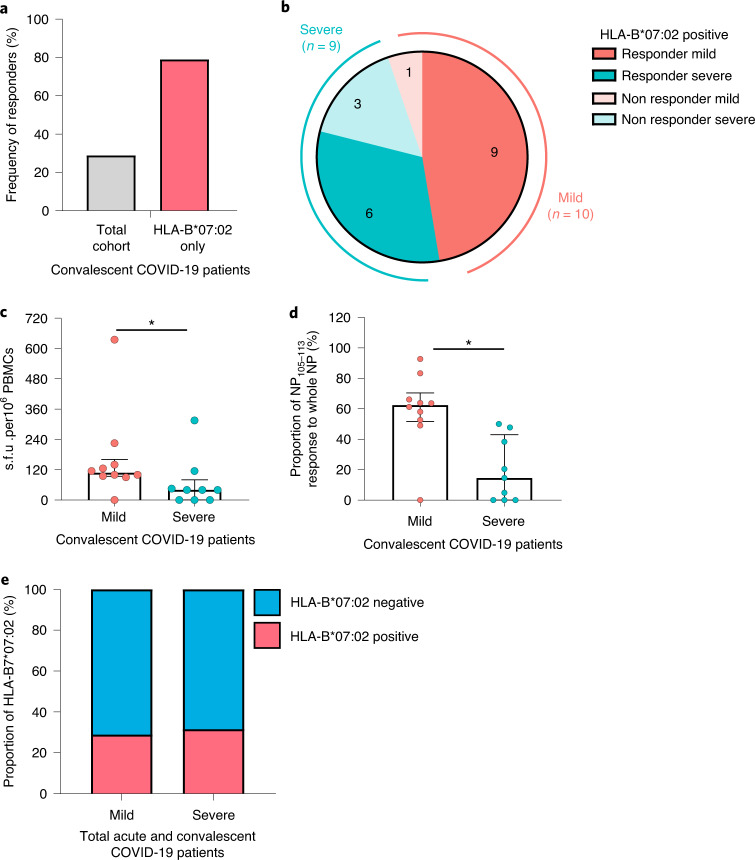
Frequency and magnitude of response to the NP_105–113_-B*07:02 epitope in patients with COVID-19. **a**, Frequency of convalescent patients with COVID-19 (*n* = 52 total patient cohort, *n* = 19 HLA-B*07:02-positive patients only) with T cells responding to NP_105–113_-B*07:02 epitope stimulation. **b**, Frequency of HLA-B*07:02-positive responders (*n* = 15) and nonresponders (*n* = 4) with mild or severe COVID-19 disease. **c**, Comparison of the magnitude of the response to the NP_105–113_ epitope between HLA-B*07:02-positive convalescent patients with COVID-19 (*n* = 10 mild, *n* = 9 severe). **d**, Proportion of NP_105-113_-specific response to overall NP response (*n* = 10 mild, *n* = 9 severe). **e**, Proportion of HLA-B*07:02 individuals compared with combined total acute and convalescent COVID-19 patients (*n* = 77 acute, *n* = 52 convalescent). Data are presented as medians with interquartile ranges (IQRs) (**c** and **d**). The Mann–Whitney *U*-test was used for analysis and the two-tailed *P* value was calculated: *P < 0.05. s.f.u., spot-forming units.

**Extended Data Fig. 1:**
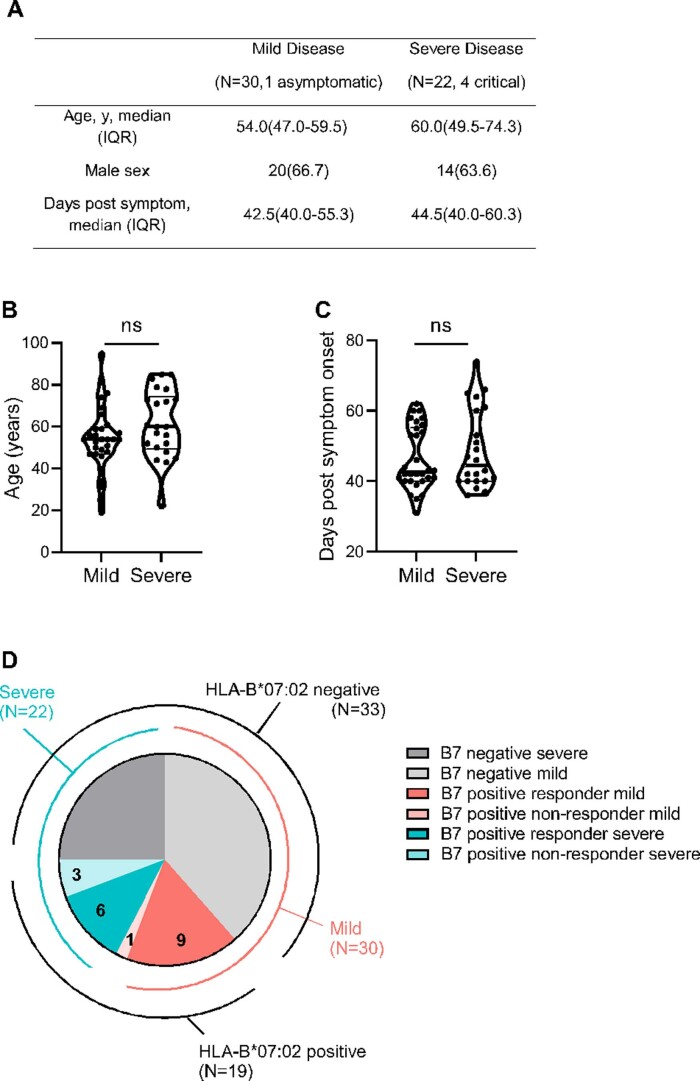
Patient characteristics. (**a**) Distribution of age, gender and days post symptom onset when sampling of SARS-CoV-2 infected patients. (**b**) and (**c**) Comparison of age (ns, *P* = 0.116) and days post symptom (ns, *P* = 0.5711) when sampling between the patient groups with mild and severe symptoms. Unpaired t-test with Welch’s correction and Mann-Whitney test were used for data analysis of (B) and (C) respectively. Two tailed *P* value was calculated. (**d**) Breakdown of patient numbers and severity; HLA-B*07:02 negative (mild N = 20 light grey, N = 13 dark grey); HLA-B*07:02 positive responders (mild N = 9 red, severe N = 6 blue); HLA-B*07:02 positive non-responders (mild N = 1 light red, severe N = 3 light blue).

A previous study has identified five dominant CD8^+^ epitopes targeting NP, including the most dominant epitope NP_105–113_ (amino acid sequence SPRWYFYYL) restricted by HLA-B*07:02 (ref. ^3^). This present study includes 52 individuals who recovered from COVID-19, comprising 30 mild cases and 22 severe cases (including 4 with critical illness; clinical features summarized in Supplementary Table 1 and Extended Data Fig. 1a–c). All the patients were HLA typed and 19 (36.5%) were HLA-B*07:02 positive (10 mild and 9 severe cases; Extended Data Fig. 1d). We proceeded to carry out ex vivo interferon (IFN)-γ ELISpot assays using HLA-B*07:02-positive convalescent samples 1–3 months postinfection. Of HLA-B*07:02 individuals, 79% (15/19) showed responses to this epitope, which accounted for 29% of individuals from the overall cohort (15/52) (Fig. 1a), including 90% (9/10) of individuals recovered from mild and 67% (6/9) from severe disease (Fig. 1b). This further confirms the dominance of this NP_105–113_-B*07:02 T cell response in our cohort, in particular in individuals recovered from mild illness. In addition, individuals recovered from mild disease made significantly stronger responses to this epitope, compared with those who had recovered from severe disease (Fig. 1c; *P* = 0.04). We also observed that this NP_105–113_-B*07:02-specific response is dominant in mild cases and makes up 60% of overall NP responses of each individual, whereas, in severe cases, the proportion is substantially lower, with an average of 19.5% (Fig. 1d; *P* = 0.015). In addition, we did not find HLA-B*07:02 association with disease outcome in our study cohorts (Fig. 1e; 77 acute and 52 convalescent patients). Our data highlight the association of the strength of this dominant epitope-induced T cell response with mild disease outcome and provide evidence that this link is epitope specific rather than a wider allelic association with HLA-B*07:02.

### Strong cytotoxicity and inhibitory receptor expression are associated with disease severity

To explore the mechanisms underlining this association, we sorted NP_105–113_-B*07:02-specific T cells at a single-cell level with peptide major histocompatibility complex class I (MHC-I) pentamers using flow cytometry. We performed single-cell analysis using SmartSeq2 for peripheral blood mononuclear cell (PBMC) samples from four convalescent patients, including two who recovered from mild COVID-19 infection (C-COV19-005, age 56 years and C-COV19-046, age 76 years) and two who recovered from severe disease in early infection (C-COV19-038, age 44 years and C-COV19-045, age 72 years). TCR sequences and transcriptomic profiles of each single cell were analyzed.

**Fig. 2:**
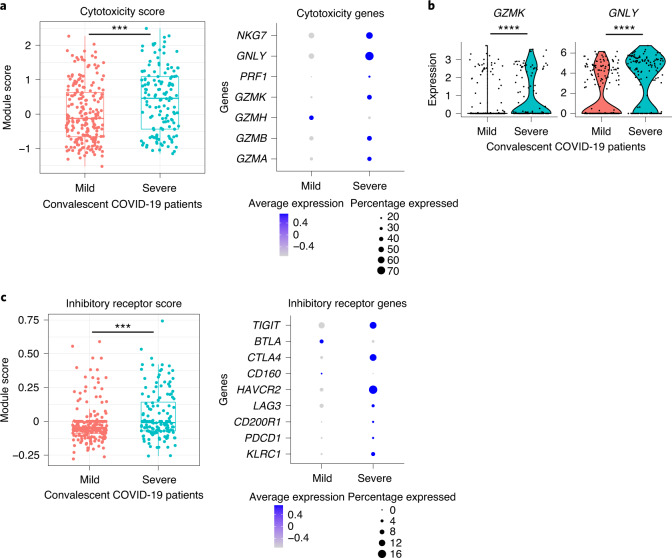
Characterization of response to the NP_105–113_-B*07:02 epitope in convalescent HLA-B*07:02-positive patients with mild and severe COVID-19. **a**, Gene sets scored based on single-cell gene expression from a SmartSeq2 RNA-seq dataset comprising two mild and two severe convalescent HLA-B7*07:02-positive patients with COVID-19 (*n* = 208 cells from mild cases, *n* = 140 cells from severe cases). Scores for cytotoxic gene expression are shown in the box plot (*P* = 0.00032). Individual genes in the cytotoxic gene set are shown on the right. **b**, Violin plots showing specific expression of cytotoxic genes *GZMK* (*P* = 3.02 × 10^−^^5^) and *GNLY* (*P* = 1.41 × 10^−^^9^). **c**, Box plot showing scores for inhibitory receptor gene set (*n* = 208 cells from mild cases, *n* = 140 cells from severe cases); right: individual genes in gene module. For all box plots, the lower and upper hinges represent the 25–75th percentiles, the central line represents the median, and the whiskers extend to the maximum and minimum values that are no greater than 1.5× the IQR. The Mann–Whitney *U*-test was used for analysis and the two-tailed *P* value was calculated: ***P < 0.001, ****P < 0.0001.

Analysis of single-cell RNA-sequencing (scRNA-seq) data with UMAP visualization and unbiased clustering revealed a homogeneous cell population; therefore we compared gene expression of CD8^+^ NP-specific, sorted single cells isolated from mild (*n* = 208 from 2 patients) and severe cases (*n* = 140 from 2 patients) by scoring expression levels of manually defined gene sets (Supplementary Table 2). Gene signatures associated with T cell cytotoxicity and inhibitory receptors were analyzed and compared between severity groups. We found that cells from patients who had recovered from severe COVID-19 have significantly higher cytotoxicity gene expression scores (Fig. 2a; *P* = 0.00032), with upregulation of *GZMK* (*P* = 3.02 × 10^−5^) and *GNLY* (*P* = 1.41 × 10^−9^) (encoding granzyme K and granulysin, respectively) (Fig. 2b). These cells also displayed increased inhibitory receptor expression (Fig. 2c; *P* = 0.00072), such as *TIGIT*, *CTLA4* and *HAVCR2* (TIM3). This supports findings published by us and others^3,11^ where patients with severe COVID-19 disease have been exposed to higher antigen loads, and that these cells are still present at 1–3 months convalescence, rather than CD8^+^ central memory T cells.

### NP_105–113_-B*07:02-specific T cells have a highly diverse TCR repertoire

**Fig. 3:**
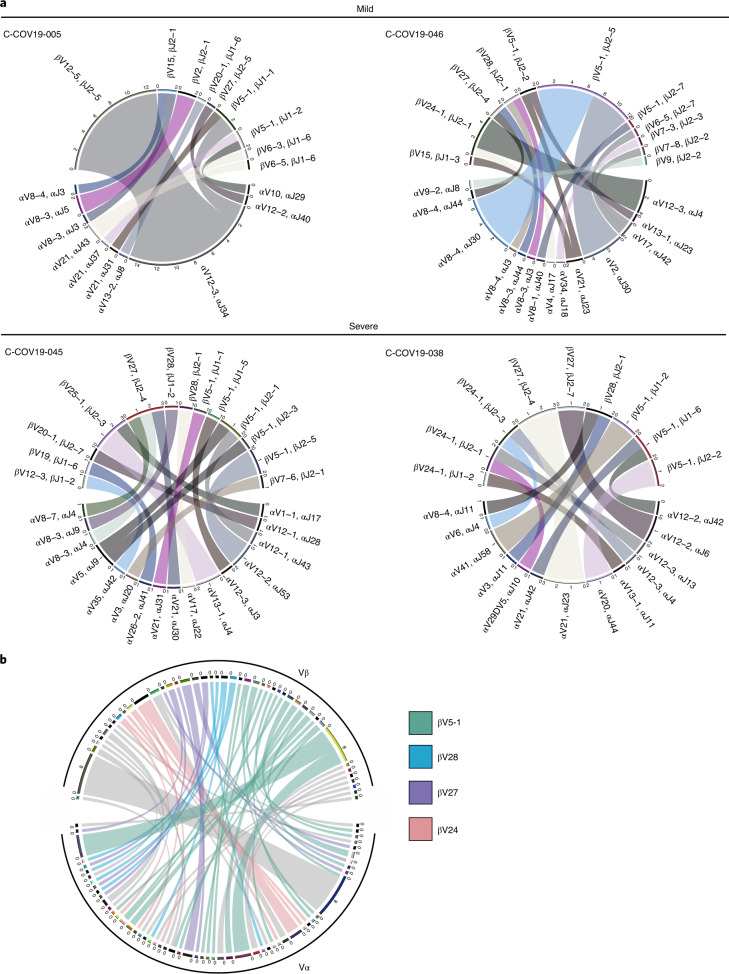
Paired αβ TCR repertoire of NP_105–113_-B*07:02-specific T cells from convalescent patients with COVID-19. **a**, Circos plots for each patient depicting αβ VJ gene usage: two patients with mild disease (C-COV19-005 and C-COV19-046) and two with severe symptoms (C-COV19-045 and C-COV19-038). **b**, Circos plot to show TCR clonotypes for all patients (clonotype defined as patient-specific V-gene usage and CDR3 amino acid sequence for Vα and Vβ). Each line represents a unique clonotype. Clonotypes that have dominant Vβ gene usage (TRBV5-1, TRBV28, TRBV27 and TRBV24) are highlighted; all others are shown in gray.

Consistent with findings by other studies^7,10^, we found that NP_105–113_-B*07:02-specific T cells from our cohort showed very broad TCR repertoires. Circos plots show paired TCR α and β chains (V and J gene usage) from the four individuals analyzed with SmartSeq2 scRNA-seq (Fig. 3a), and the combined TCR repertoire of all four patients represented by the TCR clonotype (defined separately for each patient combining V gene and CDR3 amino acid sequence) (Fig. 3b). Although the NP_105–113_-specific TCR repertoire is diverse, with unique pairings of Vα and Vβ genes, we observed that 15/45 (33.3%) of unique Vβ clonotypes were paired with several distinct Vα clonotypes. By contrast, there is only 1/55 (1.8%) Vα clonotype that pairs to multiple Vβ clonotypes; this highlights the importance of studying Vβ in the TCR repertoire. Further detailed TCR information can be found in Supplementary Table 3.

### CDR3β sequences from patients with mild COVID-19 display higher similarity to naive precursors

Several studies have reported that pre-existing, cross-reactive T cells to SARS-CoV-2 can be detected in unexposed individuals, and these T cells may have resulted from previous human seasonal coronavirus infection^7,10,12,13^. These studies found TCRs specific to NP_105–113_-B*07:02 in SARS-CoV-2-unexposed and -infected individuals. These cells were revealed as likely to be naive^7^; this is very different from the central/effector memory phenotype of SARS-CoV-2-specific T cells reported earlier. To investigate this further, we sought to determine what role these T cells might play in the early stages of SARS-CoV-2 infection and COVID-19 disease, and if these cells contribute to the association of mild disease due to their specificity for this NP-dominant epitope.

To take advantage of the results from our SmartSeq2 scRNA-seq, we first compared TCR sequences from our four convalescent patients with COVID-19 with prepandemic TCR sequences from healthy donors, published by Lineburg et al.^10^, Nguyen et al.^7^ and another study cohort, COMBAT^14^. The COMBAT dataset represents a comprehensive multi-omic blood atlas encompassing acute patients with varying COVID-19 severity (41 mild and 36 severe), and 10 healthy volunteers (prepandemic), using bulk TCR sequencing and CITE-Seq, which combines single-cell gene expression and cell-surface protein expression. TCR sequences from the Lineburg and Nguyen datasets have been experimentally validated to be specific for the NP_105–113_ epitope; however, for the COMBAT dataset, we used GLIPH2 analysis^15^ to extract TCRs with predicted specificity to this epitope based on convergence with known NP_105–113_-specific TCRs.

**Fig. 4:**
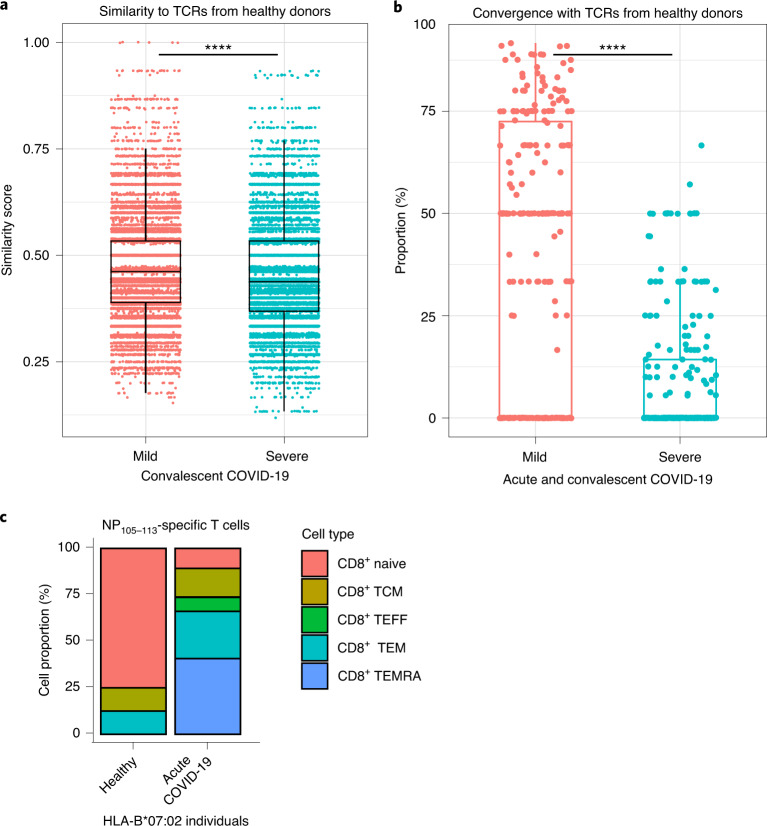
Comparison and characterization of NP_105–113_-B*07:02-specific TCRs from acute and convalescent cases of COVID-19. **a**, Similarity scores from pairwise comparisons between TCRs from prepandemic individuals (237 TCRs) and 85 unique clonotypes from convalescent patients with COVID-19 (38 mild TCRs versus 47 severe TCRs; *P* < 2.20 × 10^−^16). **b**, Proportion of acute and convalescent TCRs from patients with mild and severe COVID-19 found in the same GLIPH2 convergence groups as TCRs from 12 healthy donors (from a total of 738 TCRs from 12 mild patients, 133 TCRs from 7 severe patients and 261 TCRs from healthy individuals in 264 NP_105–113_-B*07:02-predicted convergence groups; mild versus severe: *P* < 2.20 × 10^−16^). Each dot on the graph represents a percentage for mild/severe TCRs found in a single convergence group. **c**, Breakdown of CD8^+^ T cell subtypes of T cells with predicted NP_105–113_-B*07:02 specificity from one HLA-B7*07:02-positive donor (8 cells) and HLA-B*07:02-positive patients with COVID-19 at acute stage (130 cells from 17 patients with COVID-19). TCM, T central memory; TEFF, T effector; TEM, T effector memory; TEMRA, T effector memory re-expressing CD45RA. For all box plots, the lower and upper hinges represent the 25–75th percentiles, the central line represents the median, and the whiskers extend to maximum and minimum values that are no greater than 1.5× the IQR. The Mann–Whitney *U*-test was used for analysis and the two-tailed *P* value was calculated: ****P < 0.0001.

We sought to compare NP-specific TCRs from COVID patients and healthy individuals using two different methodologies. First, we calculated similarity scores for CDR3β amino acid sequences between pairwise combinations of SmartSeq2 TCRs and prepandemic/healthy TCRs. A similarity score of 1 indicates that the pair of CDR3β sequences are identical, whereas a score of 0 indicates complete dissimilarity. In our convalescent patient cohort, CDR3β from patients with mild disease are more similar to TCRs from prepandemic/healthy individuals, than those from severe patients (Fig. 4a; *P* < 2.20 × 10^−16^). Second, we looked at the proportion of TCR sequences from patients with mild and severe disease (acute cases from COMBAT dataset and convalescent cases from previously described SmartSeq2 patients) that can be found in the same convergence groups as sequences from healthy donors, indicating high CDR3β similarity. Convergence groups containing TCRs from healthy donors appear to contain higher proportions of TCRs from mild cases rather than severe, signifying greater similarity between TCRs from prepandemic individuals and patients with mild disease (Fig. 4b; *P* < 2.2 × 10^−16^).

We were able to link predicted NP_105–113_-B*07:02TCRs with their corresponding single-cell data from the COMBAT dataset (healthy and acute SARS-CoV-2-infected patients). In this way, we could extract single-cell CITE-seq information from the COMBAT dataset, subsetted specifically to cells with predicted NP_105–113_ specificity. Cellular subtyping of these CD8^+^ NP_105–113_-B*07:02 T cells show a higher proportion of naive T cells in one HLA-B*07:02 healthy individual compared with predominantly T effector memory subtypes in patients with acute COVID-19 (*n* = 17, Fig. 4c). Overall, our data support the report that T cells bearing TCRs specific to NP_105–113_-HLA-B*07:02 in SARS-CoV-2-unexposed individuals are unlikely to have resulted from previous seasonal coronavirus infection^7^. This reinforces the finding that only NP_105–113_-B*07:02-specific T cells from acute HLA-B*07:02-positive patients are exposed to antigen and undergo T cell differentiation, whereas NP_105–113_-specific T cells in prepandemic individuals are naive precursors rather than memory cells from a previous crossreactive infection.

### Broad range and high functional avidity are associated with clonotype expansion in mild disease

**Extended Data Fig. 2:**
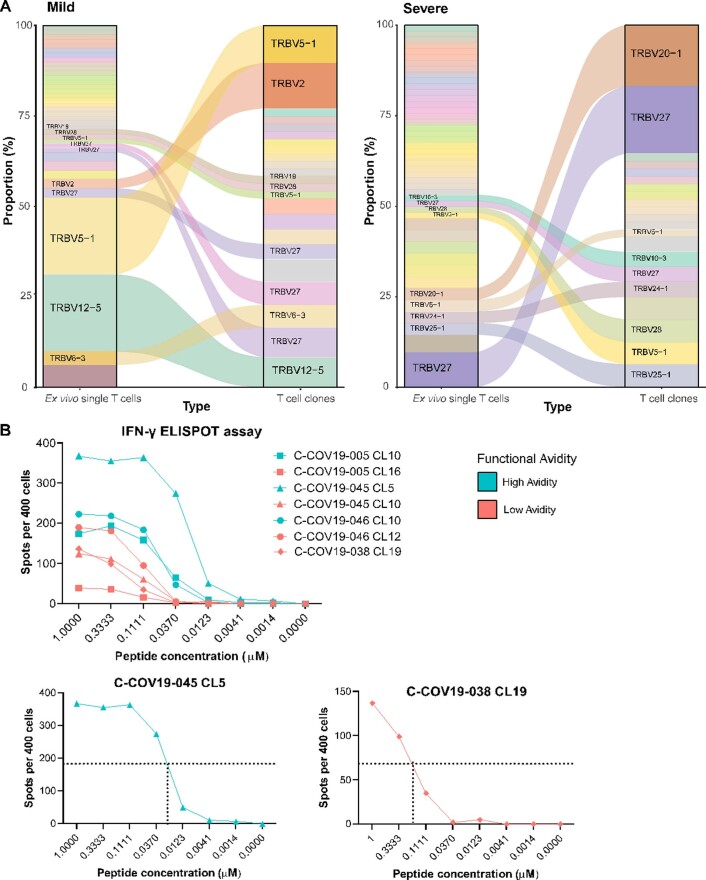
TCR clonotypes for single cells and T cell clones and EC50 derivation. (**a**) Flow diagrams to show T cell clonotypes (defined as TRBV gene usage and CDR3β sequence) between SmartSeq2 sequenced *ex vivo* single cells *(ex vivo* single T cells column) and bulk TCR sequenced T cell clones grown *in vitro* culture (T cell clones column). Panel on the left shows grouped TCRs from patients with mild disease, panel on the right for patients with severe disease. (**b**) Upper panel: IFN-γ ELISPOT assay for representative high and low functional avidity clones for each patient in blue and red respectively (C-COV19-038 only has low functional avidity clone shown). Lower panels: high functional avidity clone from C-COV19-45 and low functional avidity clone from C-COV-038 with example of EC50 derivation.

In parallel with single-cell sorting for SmartSeq2, we also sorted, cloned and expanded NP_105–113_-B*07:02-specific T cells from the same convalescent patients with COVID-19 in vitro^16,17^ to obtain pure clonal T cell populations^16,17^. We sequenced TCRs from each T cell clone with paired TCR α-chain and β-chain of each clone listed in Supplementary Table 4. When comparing the TCR sequences between T cell clones and ex vivo single cells, in vitro expanded T cell clones are a good representation for the T cells isolated for ex vivo single-cell analysis, with expanded TCRs from ex vivo single cells present as dominant TCRs from the T cell clones (Extended Data Fig. 2a).

**Fig. 5:**
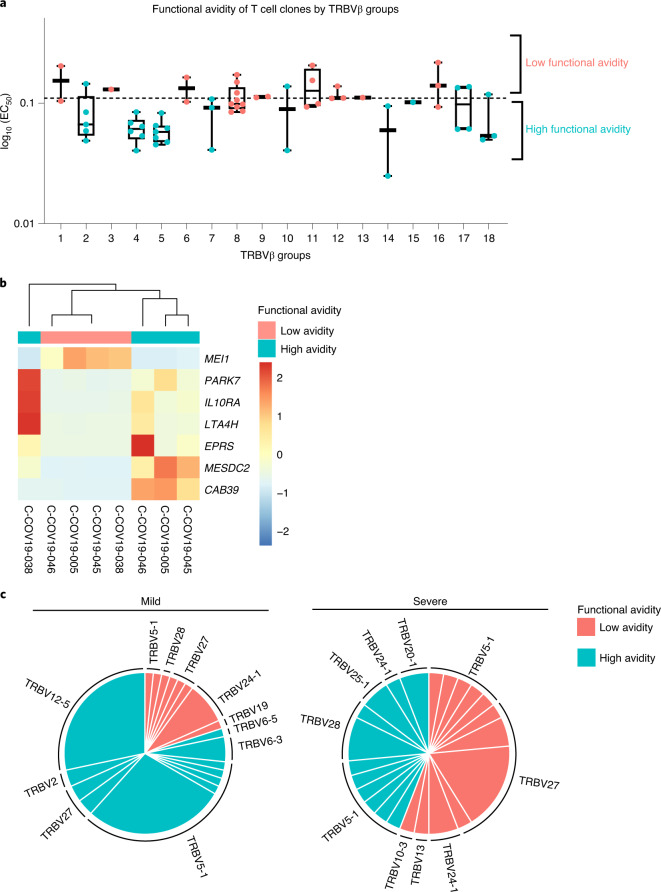
Functional avidity and clonotype expansion of NP_105–113_-B*07:02-specific T cells. **a**, Functional avidity of T cell clones by TRBV groups. NP_105–113_-B*07:02-specific T cell clones (*n* = 60) were derived from four convalescent patients with COVID-19 and functional avidity (EC_50_) was measured by IFN-γ ELISpot assay. On the basis of their *TRBV* gene usage and CDR3β sequences, T cell clones and their single-cell counterparts were sorted into 18 distinct groups, and further divided into high or low functional avidity groups (cut-off EC_50_ = 0.11). The lower and upper hinges of the box on box plots represent the 25–75th percentiles, the central line represents the median, and the whiskers extend to the maximum and minimum values. **b**, Heatmap showing differential gene expression comparing ‘pseudobulk’ high and low functional avidity single cells (88 high-avidity cells and 52 low-avidity cells; the genes shown have adjusted *P* < 0.05). **c**, Comparison of functional avidity and expansion of TCR clonotypes (defined as *TRBV* gene usage and CDR3β sequence in each patient) in convalescent patients with mild and severe COVID-19 (*n* = 4).

To provide a link between T cell clones and single-cell data by their respective TCR sequences, we divided all the T cells, including T cell clones and single cells from SmartSeq2, into 18 groups according to their unique human T cell receptor β variable (*TRBV*) gene usage and CDR3β sequence (Table 1). T cell functional avidity was measured by IFN-γ ELISpot and calculated from the half-maximal effective concentration (EC_50_) (Extended Data Fig. 2b and Supplementary Table 5). We found evidence for low and high functional avidity groups (Fig. 5a) based on the EC_50_ of T cell clones, with EC_50_ < 0.11 considered to be high-avidity and EC_50_ > 0.11 low-avidity T cells. We then aggregated RNA counts from single cells (pseudobulk) to compare differences in gene expression between the two avidity groups. Although there were only seven significantly differentially expressed genes (Fig. 5b), possibly as a result of small sample sizes and patient variation, differentially expressed genes of note upregulated in high functional avidity cells include *IL10RA*, *PARK7* and *LTA4H*. The interaction of interleukin (IL)-10 with IL-10 receptor subunit α (IL10RA) expressed on CD8^+^ T cells has been reported to directly decrease CD8^+^ T cell antigen sensitivity in patients with chronic hepatitis C infection^18^, whereas Parkinson’s disease protein 7 (PARK7) promotes survival and maintains cellular homeostasis in the setting of intracellular stress^19^. Leukotriene A4 hydrolase (LTA4H) is an enzyme with known potent anti-inflammatory activity, which functions as an aminopeptidase to degrade a neutrophil chemoattractant Pro-Gly-Pro (PGP) to facilitate the resolution of neutrophilic inflammation and prevent prolonged inflammation with exacerbated pathology and illness^20^. This supports the idea that high functional avidity T cells undergo stronger antigen stimulation and would therefore start expressing immune-dampening molecules. We further found that patients with mild disease show an increased proportion of high functional avidity TCR clonotypes, which are also more expanded than low functional avidity TCR clonotypes (Fig. 5c), whereas TCR clonotypes from patients with severe disease show equal expansion between high and low functional avidity TCRs. Therefore, the preferential expansion of high functional avidity TCR clonotypes may contribute to mild disease after SARS-CoV-2 infection.

**Table 1:** Groups defined by shared *TRBV* gene usage and CDR3β sequence between bulk TCR sequencing from T cell clones and single-cell TCR sequencing from ex vivo T cells

Group	CDR3β	*TRBV*	*TRBJ*	Functional avidity
1	CAISEPGTSGGAILDTQYF	TRBV10-3	TRBJ2-3	Low
2	CASGPATSAEQETQYF	TRBV12-5	TRBJ2-5	High
3	CASSILQGLGGSNQPQHF	TRBV19	TRBJ1-5	Low
4	CASSVLPGPPRGEQFF	TRBV2	TRBJ2-1	High
5	CSAQVGGNYNSPLHF	TRBV20-1	TRBJ1-6	High
6	CATSDLVTSGDEQFF	TRBV24-1	TRBJ2-1	Low
7	CASSGLTSLADTQYF	TRBV25-1	TRBJ2-3	High
8	CASSLITGGAKNIQYF	TRBV27	TRBJ2-4	Low
9	CASSPIAGGRKNIQYF	TRBV27	TRBJ2-4	Low
10	CASSPLTGSAERKETQYF	TRBV27	TRBJ2-5	High
11	CASSPLVGERFRKETQYF	TRBV27	TRBJ2-5	Low
12	CASSSLLAGGFYEQFF	TRBV27	TRBJ2-1	Low
13	CASSPIETAKNIQYF	TRBV28	TRBJ2-4	Low
14	CASSSITTTGAKDGYTF	TRBV28	TRBJ1-2	High
15	CASSLAGAEAFF	TRBV5-1	TRBJ1-1	High
16	CASSLAGGPLHEQFF	TRBV5-1	TRBJ2-1	Low
17	CASSSYPGLAPVQETQYF	TRBV5-1	TRBJ2-5	High
18	CASSYLPAGSSYNSPLHF	TRBV6-3	TRBJ1-6	High

### The strength of T cells responding to naturally processed epitope correlates with their functional avidity

**Fig. 6:**
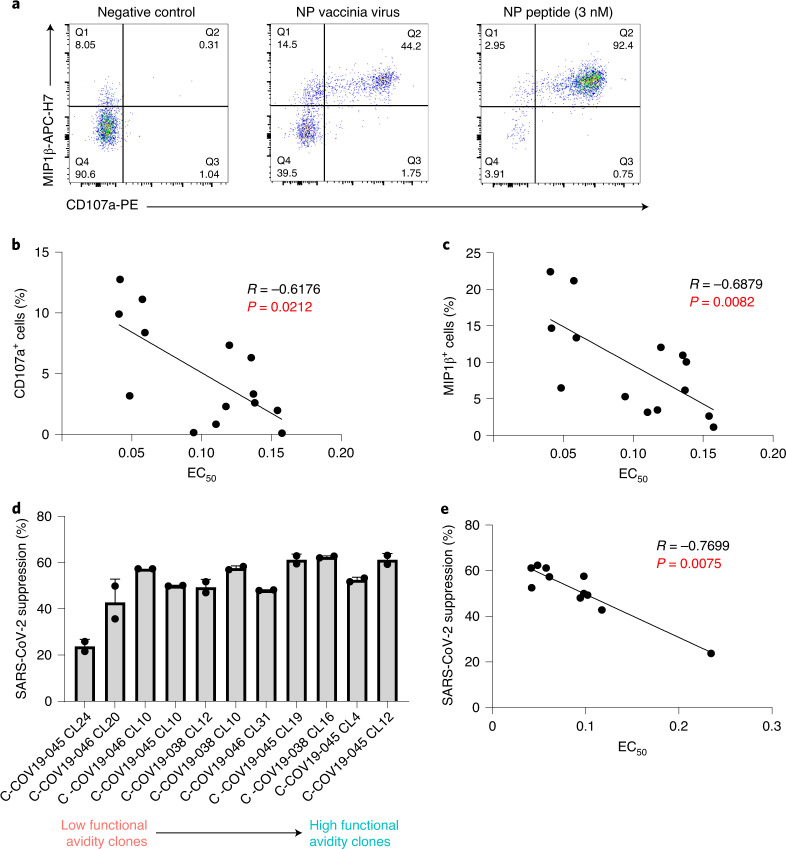
Correlation between functional avidity and antiviral efficacy in T cell clones. **a**, Representative ICS flow cytometry plots measuring MIP1β and CD107a expression on T cell clones incubated with vaccinia virus encoding NP or peptide-loaded (3 nM peptide) antigen-presenting cells. **b**, Correlation plot between CD107a expression on T cell clones incubated with NP-expressing vaccinia virus and their respective EC_50_ values (*n* = 14, *R* = −0.6176, *P* = 0.0212). **c**, Correlation plot between MIP1β production of T cell clones incubated with NP-expressing vaccinia virus and their respective EC_50_ values (*n* = 14, *R* = −0.6879, *P* = 0.0082). **d**, Inhibition of SARS-CoV-2 virus replication (Victoria strain) by T cell clones with different EC_50_ values and differing functional avidity (*n* = 11). **e**, Correlation plot between percentage of viral suppression by specific T cell clone and its corresponding EC_50_ value (*n* = 11, *R* = −0.7699, *P* = 0.0075). Spearman’s rank correlation coefficient was used for correlation analysis with the two-tailed *P* value. Bar graph is presented as mean ± s.d.

**Extended Data Fig. 3:**
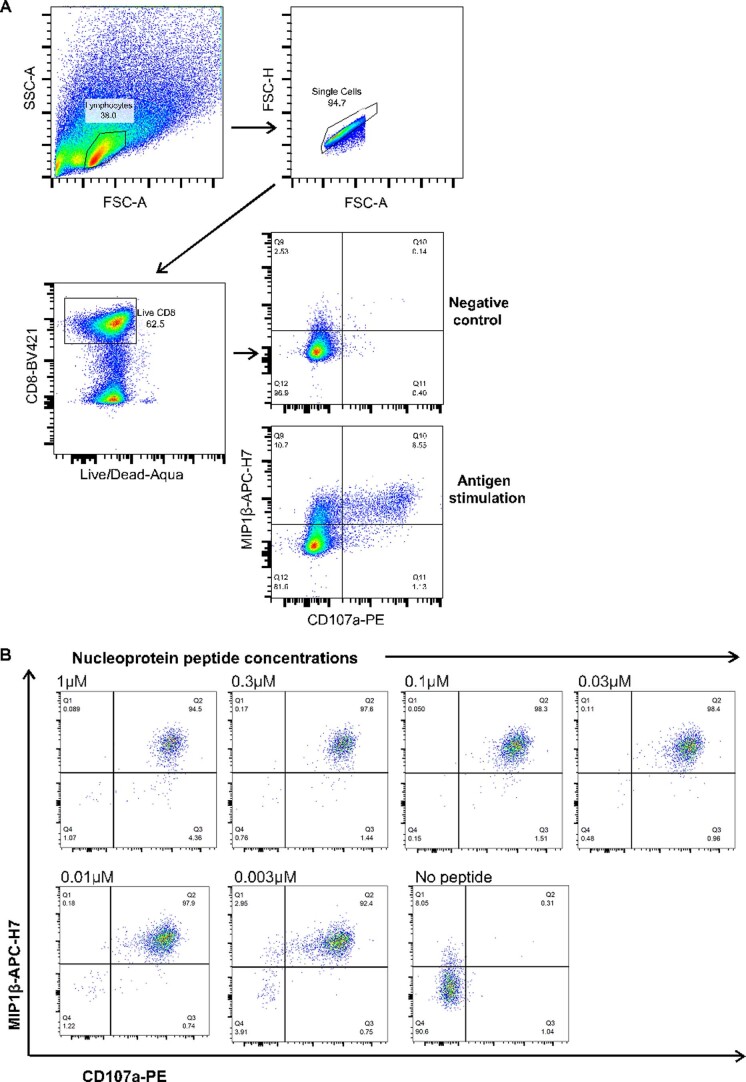
FACS gating strategy and peptide titration. (**a**) Gating strategy for flow cytometric analyses. (**b**) Nucleoprotein peptide titration at varying concentrations for ICS for MIP1β and CD107a. Representative flow cytometry plots shown (from single T cell clone).

Numerous studies including our own have shown the importance of antigen processing and presentation to T cell recognition of its antigen^21,22^. Some T cell epitopes may not be processed and presented as efficiently as others, which will subsequently diminish the T cell response to the epitope. To investigate T cell responses to naturally processed and presented viral epitopes, we made vaccinia virus-expressing SARS-CoV-2 viral proteins. We infected autologous Epstein–Barr virus (EBV)-transformed B cell lines (BCLs) with vaccinia virus-expressing NP and cocultured with NP_105–113_-B*07:02-specific T cell clones. T cell degranulation and cytokine production (CD107a expression and macrophage inflammatory protein (MIP)1β chemokine production, respectively) were then assessed by intracellular staining after 6 h of incubation (Fig. 6a). Gating for CD107a- and/or MIP1β-producing cells was based on corresponding negative controls (Extended Data Fig. 3a). When compared with the peptide-loaded targets, we found that the response to vaccinia virus-infected BCLs was much weaker, consistent with lower antigen loads. The loading of this naturally processed and presented epitope was equivalent to no more than 3 nM peptide (Extended Data Fig. 3b). Nevertheless, NP vaccinia virus-incubated clones with high CD107a expression showed a negative correlation with their individual EC_50_ values (Fig. 6b; Speaman’s rank correlation coefficient (*R*) = −0.6176, *P* = 0.0212), consistent with higher functional avidity resulting in more effective T cell killing. A similar negative correlation was also observed with MIP1β-producing cells (Fig. 6c; *R* = −0.6879, *P* = 0.0082).

To further investigate the antiviral activity of NP_105–113_-B*07:02-specific T cells, we established an in vitro SARS-CoV-2 infection system. Briefly, the angiotensin-converting enzyme 2 (*ACE2*) gene was delivered into autologous EBV-transformed BCLs by lentiviral transduction to enable SARS-CoV-2 infection via ACE2 protein expressed on the cell-surface. ACE2^+^ BCLs were purified by flow sorting and maintained by antibiotic selection, after which cells were subsequently used for SARS-CoV-2 virus infection (Victoria strain). After 48 h of incubation, intracellular viral copies were quantified by quantitative (q)PCR, where the reduction of virus replication is calculated as a percentage of virus suppression by T cells (Fig. 6d). We found that the percentage of virus suppression was strongly correlated with their functional avidity (Fig. 6e; *R* = −0.7699, *P* = 0.0075). Therefore, high functional avidity T cells can efficiently inhibit viral replication.

### NP_105–113_-B*07:02-specific T cells are maintained with preserved antiviral efficacy

#### Six months after infection

**Fig. 7:**
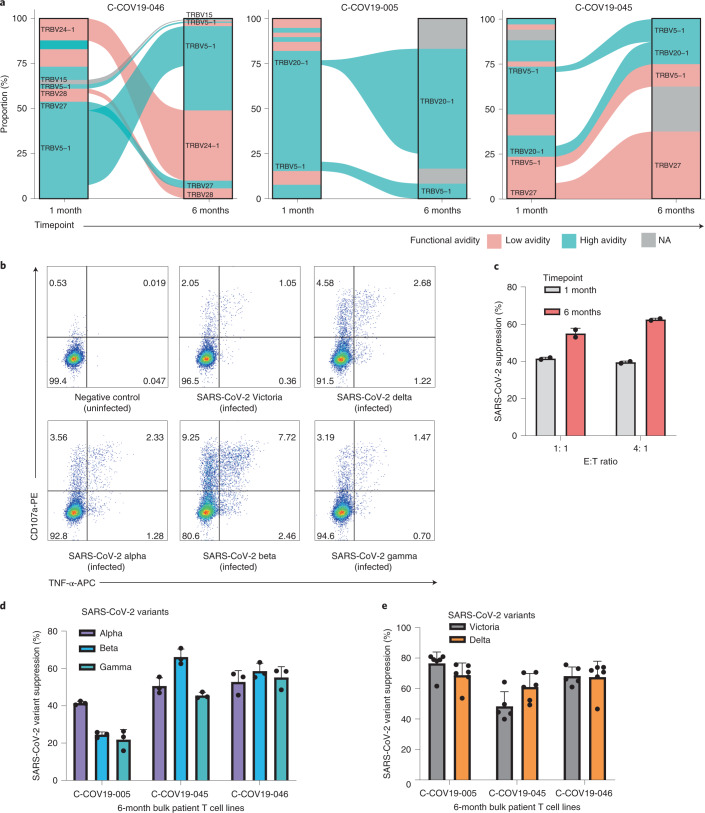
Characterization of NP_105–113_-B*07:02-specific T cell responses at 6 months convalescence. **a**, TCR repertoires of three patients at 1 month and 6 months convalescence. *TRBV* gene usage of common and expanded TCR clonotypes (defined as *TRBV* and *TRBJ* gene usage) are labeled for clarity. TCR clonotypes colored pink are low functional avidity and blue ones depict high functional avidity; clonotypes colored gray do not have similar TCRs to T cell clones. C-COV19-46 6-month cells were sequenced by 10× single-cell sequencing, and C-COV19-005 and C-C0V19-045 by bulk TCR sequencing. NA, not available. **b**, Representative ICS flow cytometry plots measuring TNF-α and CD107a expression on bulk NP_105–113_-specific T cell lines from C-COV19-046 incubated with SARS-CoV-2 Victoria, Alpha, Beta, Gamma or Delta variant-infected BCLs. **c**, Inhibition of SARS-CoV-2 viral replication (Victoria strain) by C-COV19-046 bulk NP_105–113_-specific T cell lines from 1-month (gray bars) and 6-month (red bars) convalescent samples (*n* = 2 biological replicates). Data are shown as mean ± s.d., representing three independent experiments with similar results. **d**, Antiviral activity of NP_105–113_-specific bulk T cells from 6 months convalescence against SARS-CoV-2 VOCs: Alpha (purple bars), Beta (blue bars) and Gamma (green bars) (*n* = 3 biological replicates). Data are shown as mean ± s.d., representing three independent experiments with similar results. **e**, Antiviral activity of NP_105–113_-specific bulk T cells from 6 months convalescence against SARS-CoV-2 VOCs: Victoria strain (gray bars) and Delta variant (orange bars) (*n* = 6 biological replicates). Data are shown as mean ± s.d., representing two independent experiments with similar results.

**Extended Data Fig. 4:**
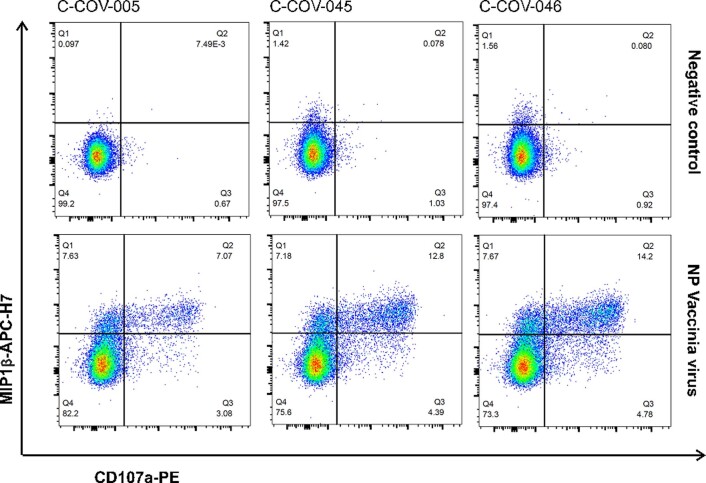
T cell response six months post infection to NP Vaccinia virus. Representative ICS flow cytometry plots showing MIP1β and CD107a protein expression after incubation with NP-expressing Vaccinia virus in three patient-derived bulk T cell lines from six months convalescent PBMC samples.

**Extended Data Fig. 5:**
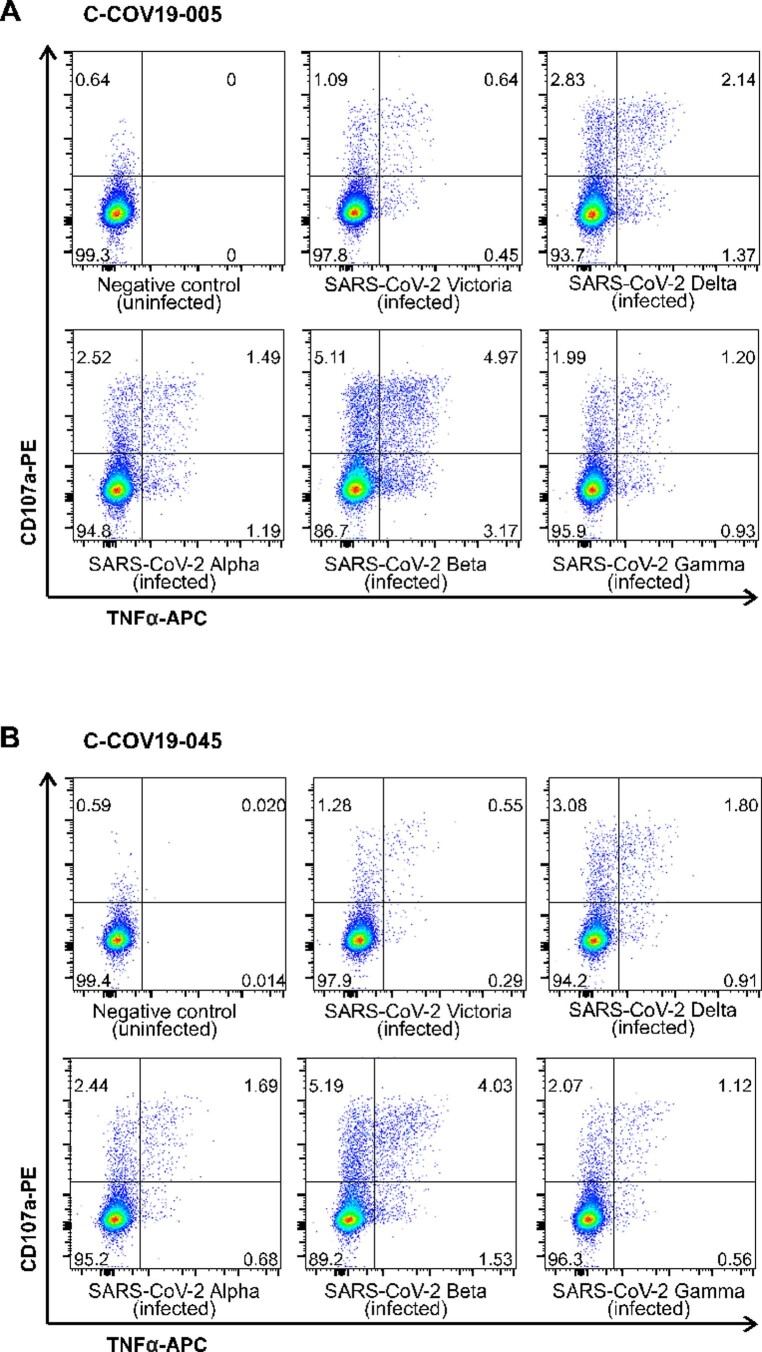
T cell response six months post infection to SARS-CoV-2 and VOCs. Representative ICS flow cytometry plots showing TNFα and CD107a protein expression in patient-derived bulk T cell lines from six months convalescent samples after incubation with SARS-CoV-2 Victoria strain and VOCs (Alpha, Beta, Gamma and Delta) infected autologous B cell lines (A) patient C-COV19-005 and (B) C-COV19-045.

To examine whether the memory T cells established postnatural infection could provide sufficient protection against secondary viral infection, we collected PBMCs from three patients (C-COV19-005, C-COV19-045 and C-COV19-046) 6 months after infection and sequenced sorted CD8^+^ NP_105–113_-B*07:02-specific T cells. We discovered that, 6 months after infection, the TCR repertoire of NP_105–113_-B*07:02-specific T cells narrows (independent of cell numbers), and the T cell memory pool contains both high and low functional avidity T cells (Fig. 7a). We then isolated and expanded further NP_105–113_-B*07:02-specific T cell bulk lines from PBMC samples taken 6 months after infection. We assessed the antiviral efficacy of these bulk T cell lines in our in vitro SARS-CoV-2 infection assays. All three T cell lines showed increased MIP1β and CD107a protein expression after incubation with NP-expressing vaccinia virus (Extended Data Fig. 4), increased tumor necrosis factor (TNF) and CD107a expression after incubation with BCLs infected with SARS-CoV-2 virus (Victoria strain) and current variants of concerns (VOCs), including the Delta variant (Fig. 7b and Extended Data Fig. 5). In addition, we found that these antigen-specific bulk cell lines are capable of suppressing SARS-CoV-2 replication (Fig. 7c) and showed strong inhibition against VOCs, including the recently emerged Alpha, Beta, Gamma and Delta SARS-CoV-2 variants (Fig. 7d,e). This is consistent with the evidence of conservation of this NP_105–113_-B*07:02 epitope, and indicates the protective role of NP_105–113_-specific T cells in secondary infection against different SARS-CoV-2 variants.

## Discussion

Our observation of strong and dominant NP_105-113_-B*07:02-specific T cell responses in mild cases highlights the possible protective role of this unique and most dominant response found so far in SARS-CoV-2 infection^3–6^. We found high similarity and convergence of TCRs in HLA-B*07:02-positive healthy and recovered individuals, with naive precursors identified in prepandemic samples supporting previous reports^7,10^. In addition, T cells from convalescent patients with mild disease show higher functional avidity as well as better effector and antiviral function compared with convalescent patients with severe COVID-19. It is interesting that the immune memory pools postinfection (6 months convalescence) are narrowed but remain proportional; we found no bias toward high or low functional avidity TCRs during immune memory contraction. Moreover, this dominant NP_105–113_-specific response restricted by HLA-B*07:02 is associated with protection against severe disease, but does not associate with HLA-B*07:02 when analyzed alone.

The highly diverse TCR repertoire of NP_105–113_-B*07:02-specific T cells in recovered individuals is of particular interest; whether this is a common phenomenon of acute primary virus infection or these responses are unique, with high frequency and broader choice of TCR precursors available, would merit future investigation. The latter is supported by our finding that TCRs in COVID-19-recovered individuals can be similar to those found in prepandemic individuals, in particular patients with mild symptoms. We hypothesize that NP_105–113_-B*07:02-specific T cell responses play an important role in protecting individuals from severe illness, which is probably due to early priming and expansion of high-frequency naive TCRs specific to this epitope.

We further provide evidence to support our hypothesis by studying a cohort of patients with acute SARS-CoV-2 infection, by analyzing the TCR repertoire in HLA-B*07:02-positive patients. We first found high frequencies of TCR precursors with naive phenotype in HLA-B*07:02-positive healthy donors; this further supports the recent findings, from Nguyen et al.^7^, that these T cell precursors bearing NP-specific TCRs are not due to pre-existing memory from seasonal coronaviruses. We observed that strong cytotoxicity and inhibitory receptor expression are associated with disease severity, where NP_105–113_-B*07:02-specific T cells are more activated and well differentiated in individuals recovered from severe illness. This is probably the result of stronger antigen stimulation and expansion during the acute phase of viral infection.

We found overall high functional avidity T cell expansion in mild cases, and that high functional avidity is associated with expression of immune-damping molecules such as IL10RA, PARK7 and LTA4H, which could potentially act to prevent prolonged inflammation with exacerbated pathology and illness^18,20,23,24^. In particular, LTA4H has a known function as an aminopeptidase to degrade a neutrophil chemoattractant PGP, facilitating the resolution of neutrophilic inflammation, which is known to be associated with immunopathology in respiratory virus infections such as COVID-19 (ref. ^25^). This further provides evidence that expansion of high avidity precursors in mild cases contributes to the overall protective immunity from severe illness.

We show that NP_105–113_-B*07:02-specific T cells can respond to cells infected with live SARS-CoV-2 virus as well as emerging viral variants, and most importantly suppress virus replication in infected cells. The magnitude and strength of the response to naturally processed epitopes presented by infected cells correlate with their functional avidity. The proportional expansion with both high and low functional avidity T cells was maintained in CD8^+^ T cell memory pools after immune memory contraction (at 6 months postinfection), and these cells could suppress virus replication efficiently for all viral variant strains. This is not surprising due to the conservation of this epitope across viral strains, and provides some reassurance that memory T cells generated from natural infection could respond to newly emerged variants and still provide protective immunity.

Taken together, we have demonstrated that, first, we found strong association of NP_105–113_-HLA-B*07:02-specific T cell response with mild disease; second, the protective effect of NP_105–113_-HLA-B*07:02-specific TCRs from severe illness may be due to early expansion of high-frequency naive T cell precursors bearing these TCRs. Moreover, we found that the TCR repertoire is not disturbed after virus infection and immune memory contraction, and that these memory T cells are able to suppress the original SARS-CoV-2 viral strain (contracted by the patient) as well as newly emerged viral strains.

We recognize that there are a number of limitations to the present study, for example, the number of convalescent patients analyzed by single-cell gene expression and TCR sequencing (*n* = 4) is small. Also, the number of NP_105–113_-B*07:02-specific cells from prepandemic donors and patients with acute COVID-19 is low, partly because these cells were not pentamer sorted before analysis. In the present study, we focus on CD8^+^ T cell responses to a single epitope; however, it may be useful in the future to see whether there are any distinct features or features shared with other dominant responses. Although our data support high-frequency naive T cell precursors probably contributing to mild disease outcome, it is also possible, as the consequence of high viral load and overstimulation caused by high functional avidity T cells (with higher proportion of precursor TCRs), leading to exhaustion and depletion during the acute virus infection, which merits further investigation, including larger cohorts sizes. We found that a higher proportion of TCR sequences from mild cases converged with those from prepandemic individuals, although it may be possible that this observation arose from higher numbers of TCRs from mild patients used as input for this convergence analysis. The specifics of antigen loading of this particular epitope, compared with other NP epitopes, as well as variation in levels of protein expression and localization, are also unknown and warrant further investigation.

## Methods

### Study participants and clinical definitions

Patients were recruited from the John Radcliffe Hospital in Oxford, UK, between March 2020 and April 2021 by identification of patients hospitalized during the SARS-CoV-2 pandemic. Patients were recruited into the Sepsis Immunomics study and had samples collected during acute disease and convalescence. Patients were sampled at least 28 d after symptom onset. Written informed consent was obtained from all patients. Ethical approval was given by the South Central-Oxford C Research Ethics Committee in England (ref. 19/SC/0296). Clinical definitions were defined as previously described^3^.

### Generating ACE2-transduced EBV-transformed BCLs

EBV-transformed BCLs were generated as described previously^26^. The complementary DNA for the human *ACE2* gene (ENSG00000130234) was cloned into a lentiviral vector that allows coexpression of enhanced green fluorescent protein and a puromycin resistance marker (Addgene, plasmid no. 17488). The plasmids were cotransfected with packaging plasmids pMD2.G and psPAX2 into HEK293-TLA using PEIpro (Polyplus). Lentiviral supernatant was collected 48 h and 72 h post-transfection and concentrated by ultracentrifugation. EBV-transformed BCLs were infected by ACE2 lentivirus at a multiplicity of infection (MOI) of 0.1 with 8 µg ml^−1^ of polybrene (Sigma-Aldrich) overnight, then washed and cultured for 3–5 d. ACE2-expressing B cells were stained using primary goat anti-human ACE2 antibody (R&D, 1:20) and donkey anti-goat AF647 secondary antibody (Abcam, 1:1,000), followed by cell sorting via flow cytometry. B cells with stable expression of ACE2 were maintained with 0.5 µg ml^−1^ of puromycin (Thermo Fisher Scientific). *Mycoplasma* testing was carried out every 4 weeks with all cell lines using MycoAlert detection kit (Lonza).

### Generating T cell lines and clones

Short-term SARS-CoV-2-specific T cell lines were established as previously described^17^. Briefly, 3 × 10^6^ to 5 × 10^6^ PBMCs were pulsed for 1 h at 37 °C with 10 μM peptides, containing T cell epitope regions and cultured in R10 (RPMI 1640 medium with 10% fetal calf serum, 2 mM glutamine and 100 mg ml^−1^ of penicillin–streptomycin) at 2 × 10^6^ cells per well in a 24-well Costar plate. IL-2 was added to a final concentration of 100 U ml^−1^ on day 3 and cultured for a further 10–14 d. T cell clones were generated by sorting HLA-B*07:02 NP_105–113_ pentamer^+^ CD8^+^ T cells at a single-cell level from thawed PBMCs or short-term cell lines. T cell clones were then expanded and maintained as described previously^27^.

### Generating vaccinia virus-expressing SARS-CoV-2 NP

SARS-CoV-2 nucleocapsid (NP) expression vectors (gifts from P. Wang, Shandong University, Shandong, China^28^) were first digested with KpnI and SacII. The resulting fragment was cloned into vaccinia virus (VACV) expression vector pSC11, which was inserted with a DNA segment encoding KpnI and SacII digestion sites (GGTACCGCGGCCGCCCGCGG). The SARS-CoV-2 NP-expressing recombinant VACV (rVACV) was produced as described previously^29–31^. In brief, HEK293T cells (American Type Culture Collection (ATCC), catalog no. CRL-11268) were transfected with 3 μg of pSC11-containing NP with poly(ethylenimine). At 24 h post-transfection, cells were infected with the Lister strain of VACV at an MOI of 1 for 48 h. Infected cells were collected for recombinant virus purification using TK143B cells (ATCC, catalog no. CRL-8303) in 25 μg ml^−1^ of bromodeoxyuridine. The NP-expressing rVACV was selected through β-galactosidase staining by supplementing 25 μg ml^−1^ of X-gal to an agarose overlay. Master stocks of rVACV were prepared by infection on rabbit RK13 (ATCC, catalog no. CCL37) and titrated on African green monkey BS-C-1 (ATCC, catalog no. CCL26) cells.

### SARS-CoV-2 live virus propagation and titration

SARS-CoV-2 Victoria 01/20 strain (BVIC01) and variants of concern—Alpha (Lineage B.1.1.7, 20I/501Y.V1.HMPP1) and Beta (Lineage B.1.351, 201/501.V2.HV001)—were originally from Public Health England and provided by J. McKeating^32^. SARS-CoV-2 Gamma (Lineage P.1) was provided by G. Screaton^33^. The Delta (B.1.617.2) variant was originally from W. Barclay and T. De Silva (G2P-UK) and provided by G. Screaton^34^. In brief, Victoria 01/20, Alpha and Beta variants were propagated with Vero E6 cells, whereas SARS-CoV-2 Gamma and Delta variants were propagated with Vero E6/TMPRSS2 (provided by A. Townsend). Naive Vero E6 or Vero E6/TMPRSS2 cells were plated overnight and infected with SARS-CoV-2 at an MOI of 0.003. Cultures were harvested when visible cytopathic effects were observed 48–72 h later. Virus-containing supernatant was aliquoted and stored at −80 °C. The viral titer was determined by plaque assay with Vero E6 or Vero E6/TMPRSS2 as previously described, and plaque-forming units per ml were used to calculate the MOI.

### IFN-γ ELISpot assay

Ex vivo IFN-γ ELISpot assays were performed using either freshly isolated, cryopreserved PBMCs or antigen-specific T cell clones as described previously^3^. For ex vivo ELISpots, peptides were added to 2 × 10^5^ PBMCs per test at 2 μg ml^−1^ for 16–18 h. When using T cell clones, autologous EBV-transformed BCLs were first loaded with peptides at threefold titrated concentrations and subsequently cocultured with T cells at an effector:target (E:T) ratio of 1:50 for at least 6 h. To quantify antigen-specific responses, data were collected with AID ELISpot 7.0, mean spots of the control wells were subtracted from the positive wells (phytohemagglutinin stimulation) and the results expressed as spot-forming units (s.f.u.) per 10^6^ PBMCs. Responses were considered positive if results were at least three times the mean of the negative control wells and >25 s.f.u. per 10^6^ PBMCs. If negative control wells had >30 s.f.u. per 10^6^ PBMCs or positive control wells were negative, the results were excluded from further analysis.

### Flow cytometric sorting of NP_105–113_-B*07:02-specific CD8^+^ T cells

NP_105–113_-B*07:02-specific CD8^+^ T cells were stained with phycoerythrin (PE)-conjugated HLA-B7 NP_105–113_ pentamer (ProImmune). Live/dead fixable Aqua dye (Invitrogen) was used to exclude nonviable cells from the analysis. Cells were washed and stained with the following surface antibodies: CD3-FITC (BD Biosciences), CD8-PercP-Cy5.5, CD14-BV510, CD19-BV510 and CD16-BV510 (BioLegend). After exclusion of nonviable/CD19^+^/CD14^+^/CD16^+^ cells, CD3^+^CD8^+^pentamer^+^ cells were sorted directly into 96-well PCR plates (Thermo Fisher Scientific) using a BD Fusion sorter or BD FACS Aria III (BD Biosciences) and stored at −80 °C for subsequent analysis.

### ScRNA-seq

ScRNA-seq with ex vivo sorted CD8^+^pentamer^+^ T cells was performed using SmartSeq2 (ref. ^35^) with the following modifications: reverse-transcription (RT) and PCR amplification were performed as described^35^ with the exception of using ISPCR primer with biotin tagged at the 5′ end and increasing the number of cycles to 25. Sequencing libraries were prepared using the Nextera XT Library Preparation Kit (Illumina) and sequencing was performed on Illumina NextSeq sequencing platform with NextSeq Control Software v.4.

### Deep sequencing of TCR repertoire of T cell clones

From each T cell clone, 1 × 10^5^ cells were harvested and washed with phosphate-buffered saline (PBS). Total RNA was extracted using RNeasy Plus Micro Kit (QIAGEN), and 100 ng of total RNA from each T cell clone was used to generate full-length TCR repertoire libraries for Illumina Sequencing using a SMARTer Human TCR a/b Profiling Kit (Takara) following the supplier’s instructions. The cDNA sequences corresponding to variable regions of TCR-α and/or TCR-β transcripts were amplified with primers, including Illumina indices, allowing for sample barcoding. PCR products were then purified using AMPure beads (Beckman Coulter). The quantity and quality of cDNA libraries were checked on an Agilent 2100 Bioanalyzer system. Sequencing was performed using MiSeq reagent Kit v.3 (600 cycles) on MiSeq (Illumina) with MiSeq Control Software v.2.6.2.1.

### Intracellular cytokine staining

Intracellular cytokine staining (ICS) was performed as described previously^3^. Briefly, T cells were cocultured with peptide-loaded or virus-infected BCLs at an appropriate E:T ratio for a 6 h incubation with GolgiPlug and GolgiStop, and surface stained with PE-anti-CD107a (1:20). Dead cells were labeled using Live/Dead Fixable Aqua dye (Invitrogen); after staining with BV421-anti-CD8 (1:40), cells were then washed, fixed with Cytofix/Cytoperm and stained with AF488-anti-IFN-γ (1:33), APC-anti-TNF-α (eBioscience, 1:500) and APC-H7-anti-MIP1β (1:33). Negative controls without peptide stimulation or virus infection were run for each sample. All reagents were from BD Bioscience unless otherwise stated. All samples were acquired on Attune NxT Flow Cytometer (software v.3.2.1) and analyzed using FlowJo v.10 software (FlowJo LLC).

### Evaluation of T cell response to vaccinia virus infection

EBV-transformed BCLs were infected with Lister strain vaccinia virus at an MOI of 3 for 90–120 min at 37 °C. Cells were washed to remove any virus and incubated overnight in R10 at 37 °C. Cells were counted and cocultured with T cells at an E:T ratio of 1:1. Degranulation (CD107a expression) and cytokine production of T cells were evaluated by ICS as described above.

### Evaluation of T cell response to live virus infection

EBV-transformed BCLs expressing ACE2 were infected with SARS-CoV-2 viruses at an MOI of 1 for 120 min at 37 °C. Cells were washed and incubated in R10 at 37 °C. After 24 h, cells were counted and cocultured with T cells at an E:T ratio of 1:1. Degranulation (CD107a expression) and cytokine production of T cells were evaluated by ICS as described above.

### Live virus suppression assay

EBV-transformed BCLs expressing ACE2 were infected with SARS-CoV-2 viruses at an MOI of 0.1 for 120 min at 37 °C. Cells were washed and cocultured with T cells at an E:T ratio of 4:1. Control wells containing virus-infected targets without T cells were also included. After 48 h incubation, cells were washed with PBS and lysed with buffer RLT (QIAGEN). RNA was extracted using RNeasy 96 kit (QIAGEN). Virus copies were quantified with Takyon Dry one-step RT-qPCR (Eurogentec) using SARS-CoV-2 (2019-nCoV) CDC qPCR Probe Assay (IDT, ISO 13485:2016) and human β_2_-microglobulin as an endogenous control (Applied Biosystems). The suppression rate was calculated by the percentage reduction of virus replication by T cells.

### SmartSeq2 scRNA-seq data processing

BCL files were converted to FASTQ format using bcl2fastq v.2.20.0.422 (Illumina). FASTQ files were aligned to human genome hg19 using STAR v.2.6.1d^36^. Reads were counted using featureCounts (subread v.2.0.0 (ref. ^37^)). The resulting counts matrix was analyzed in R v.4.0.1 using Seurat v.3.9.9.9010 (ref. ^38^).

### SmartSeq2 scRNA-seq analysis

Cells were filtered using the following criteria: minimum number of cells expressing specific gene = 3, minimum number of genes expressed by cell = 200 and maximum number of genes expressed by cell = 4,000. Cells were excluded if they expressed more than 5% mitochondrial genes. Patient-specific cells were integrated using Harmony v.1.0 to remove batch effects. The AddModuleScore function (Seurat) was used to look at the expression of specific gene sets (Supplementary Table 2). The average expression of a gene set was calculated, and the average expression levels of control gene sets were subtracted to generate a score for each cell relating to that particular gene set. Higher scores indicate that that specific signature is expressed more highly in a particular cell compared with the rest of the population. Module scores were plotted using ggplot2 v.3.3.2 (ref. ^39^).

### SmartSeq2 TCR repertoire analysis

TCR sequences were reconstructed from scRNA-seq FASTQ files using MiXCR v.3.0.13 (refs. ^40,41^) to produce separate TRA and TRB output files for analysis. The output files were parsed into R using tcR v.2.3.2. For paired αβ TCRs, cells were filtered to retain 1α1β or 2α1β cells. Circos plots showing paired αβ TCRs were created using circlize v.0.4.12 (ref. ^42^). Lists were generated for all 1β cells (regardless of number of α) to use for downstream analysis.

### Clustering

Input data for clustering was all 1β from scRNA-seq cells and 1β from bulk sequencing T cell clones. Single cells and clones were grouped by Vβ usage first; TCRs from either single cells or clones with unique Vβ gene usage were excluded. Each Vβ group was broken down into subgroups based on the CDR3β sequence; any TCRs from either single cells or clones that contained unique CDR3β sequences were excluded. Only TRBV27, TRBV28 and TRBV5-1 showed multiple CDR3β sequences with the same gene usage. After plotting EC_50_ values of T cell clones, groups were classified as low or high functional avidity based on a manually defined cut-off (EC_50_ = 0.11). This led to a list of 18 groups with unique Vβ gene usage and CDR3β sequences shared among the TCRs from single-cell sequencing and bulk T cell clone sequencing.

To group as many single cells into one of these 18 groups, the stringsim function was used (stringdist v.0.9.6 (ref. ^43^)) to compare the similarity between all SmartSeq2 CDR3β sequences and each of the 18 CDR3β from the single cell/clone grouping. A minimum similarity score of 0.7 was used to decide whether a TCR from a single cell should belong to one of the 18 groups. Once allocated, the single cell was annotated as being high or low functional avidity based on its group number.

### TCR sequencing from T cell clones (bulk sequencing)

BCL files were converted to FASTQ files as described earlier. TCRs were extracted using MiXCR and the resulting output files (TRA and TRB) were parsed into R using tcR as described earlier. TCRs were filtered to retain 1α1β for each clone. TCR clonotypes (defined as Vβ gene usage and CDR3β sequence) were compared between single TCR and bulk TCR sequencing using ggalluvial v.0.12.2 (ref. ^44^). The predicted functional avidity annotation was overlaid on to the plots using the stringsim function as previously described to classify TCRs into high or low functional avidity groups (minimum score 0.5).

### VDJ 10× sequencing

Raw BCL files were processed using 10× Genomics Cellranger v.5.0.0 (ref. ^45^). For donor deconvolution from multiplexed single-cell data, cellSNP v.0.3.2 (ref. ^46^) was used to generate a list of SNPs from Cellranger output (BAM file). Vireo v.0.5.6 (ref. ^47^) was used to demultiplex the sequencing data into individual patients from the pooled sequenced libraries, based on previously generated SNP-list TCRs from 10× sequencing representing 6-month convalescence, and were compared with 1-month convalescence TCRs (SmartSeq2) from the same patient using ggalluvial. The predicted functional avidity annotation was overlaid on to the plots using the stringsim function, as previously described, to classify TCRs into high or low functional avidity groups (minimum score 0.5).

### Gene expression analysis and cell subtyping from acute COVID-19 dataset

Normalized single-cell gene expression data for T cells from the COMBAT dataset (level 2 subsets a and b)^14^ was annotated with specific T cell subtypes according to COMBAT multimodal analysis, COMBAT TCR chain information and patient metadata. Any cells without both a CD8^+^ multimodal major cell type classification and TCR chain information were excluded from further analysis. A simplified severity grouping based on the World Health Organization’s ordinal scale, which ranges from 0 to 8 (https://www.who.int/blueprint/priority-diseases/key-action/COVID-19_Treatment_Trial_Design_Master_Protocol_synopsis_Final_18022020.pdf), was used to classify participants into the following: uninfected (0), mild (1–4), severe (5–7) or death (8).

### GLIPLH2 analysis

A GLIPH2 CD8^+^ TCR input file was created from the following datasets: COMBAT 10× paired-chain single-cell and bulk TCR from all available participants^14^; pentamer-sorted NP_105–113_-B*07:02-specific TCR sequences and clonally expanded cells used to test functional avidity processed using MiXCR (as described previously); and NP_105–113_-B*07:02-specific TCR sequences from the Lineburg and Nguyen datasets^7,10^. Clonotypes were defined as having a unique combination of CDR3β amino acid sequence, *TRBV* gene, *TRBJ* gene and CDR3α amino acid sequence. Where no or multiple CDR3α sequences were available for a cell, a not available (NA) value was used for the CDR3α field in accordance with GLIPH2 input guidelines. For each clonotype, additional information indicating dataset origin was appended as part of the ‘condition’ field. For the 10× COMBAT dataset, CD8^+^ clonotypes were distinguished from CD4^+^ clonotypes based on the multimodal classification of cells within each clone.

A matching GLIPH2 participant HLA input file was created using COMBAT formal HLA-typing data and, where no formal typing was available, from imputed HLA typing^3,14^, in addition to published HLA data relating to the Lineburg and Nguyen datasets^7,10^.

The GLIPH2 irtools.centos v.0.01 (ref. ^15^) was run on a CentOS Linux platform (release 8/3/2011) using the CD8^+^ TCR and HLA input files above, together with CD8^+^-specific V-gene usage, CDR3 length and TCR reference files from the GLIPH2 repository and using the following parameters: local_min_pvalue = 0.001; p_depth = 1000; global_convergence_cutoff = 1; simulation_depth = 1000; kmer_min_depth = 3; local_min_OVE = 10; algorithm = GLIPH2; all_aa_interchangeable = 1; number_of_hla_field = 1; and hla_association_cutoff = 0.050000. A GLIPH score summary file was then programmatically curated, identifying convergence groups containing TCRs known to be NP_105–113_-B*07:02 specific as described previously, with associated GLIPH2 scoring and HLA prediction.

Convergence groups from this file were further categorized as being associated with or lacking association with HLA-B*07:02 based on having a GLIPH2 HLA score <0.05 or ≥0.05, respectively. Only clonotypes belonging to a HLA-B*07-associated convergence group, which were from participants known to have a HLA-B*07:02 allele, were deemed to be HLA-B*07:02-positive TCRs. Any clonotypes from convergence groups lacking HLA-B*07:02 association, but belonging to patients with a HLA-B*07:02 allele, were deemed ambiguous and excluded from the HLA-B*07:02-negative clonotype set.

### Similarity between prepandemic and convalescent COVID-19 TCRs

NP_105–113_-specific TCRs from prepandemic individuals (predicted from the COMBAT dataset or experimentally defined by the Lineburg and Nguyen datasets^7,10^) were compiled to form a single list of sequences (237 TCRs). Similarity scores were calculated from pairwise comparisons between each CDR3β sequence from the prepandemic/healthy list and each CDR3β sequence from 85 unique clonotypes of 4 convalescent patients with COVID-19 (clonotype defined per patient, *TRBV* gene usage and CDR3β sequence). A score of 1 indicates total similarity whereas a score of 0 is total dissimilarity. Each score was plotted on a box plot using ggplot2.

### Pseudobulk and differential gene expression

RNA counts from SmartSeq2 single cells were aggregated into groups based on patient origin and high/low functional avidity, and converted to a Single Cell Experiment (v.1.10.1) object^48^. Differential gene expression was conducted using DESeq2 v.1.28.1 on aggregated (pseudobulk) counts. Significant genes were visualized on a heatmap using pheatmap v.1.0.12.

### Statistics

A Mann–Whitney nonparametric *U*-test was used to compare two groups (*R*); other statistical tests were carried out using GraphPad Prism. Nonlinear regression with variable slope (four parameters) in a dose–response–stimulation model was used for calculating the EC_50_ of T cell clones. Spearman’s rank correlation coefficient was used for correlation analysis. NS, not significant; *P < 0.05, **P < 0.01, ***P < 0.001, ****P < 0.0001.

### Reporting Summary

Further information on research design is available in the Nature Research Reporting Summary linked to this article.

## Online content

Any methods, additional references, Nature Research reporting summaries, extended data, supplementary information, acknowledgements, peer review information; details of author contributions and competing interests; and statements of data and code availability are available at 10.1038/s41590-021-01084-z.

## Supplementary information

Supplementary Information Supplementary Tables 2 and 6.

Reporting Summary

Peer Review Information

Supplementary Tables Supplementary Table 1. Participant characteristics. Gender, days postsymptom onset and age shown for each participant. Supplementary Table 3. αβ VJ gene usage and CDR3 sequences for ex vivo single-cell TCR sequencing. Supplementary Table 4. αβ VJ gene usage and CDR3 sequences from bulk TCR sequencing of T cell clones. Supplementary Table 5. EC_50_ values from selected T cell clones.

## Data Availability

The raw data from all the main and supplementary figures are available on request. In addition the following published datasets were used: Lineburg et al.^10^ (10.1016/j.immuni.2021.04.006), Nguyen et al.^7^ (10.1016/j.immuni.2021.04.009) and COMBAT (10.1101/2021.05.11.21256877).

## Extended data

are available for this paper at 10.1038/s41590-021-01084-z.
