# Supplementary material for: An immunodominant NP105–113-B*07:02 cytotoxic T cell response controls viral replication and is associated with less severe COVID-19 disease
Source: Nat Immunol. 2021 Dec 1;23(1):50–61. doi: 10.1038/s41590-021-01084-z (PMC8709787; doi:10.1038/s41590-021-01084-z)
Supplement: Supplementary file 1 — Supplementary Tables 2 and 6. [file 41590_2021_1084_MOESM1_ESM.pdf]

nature portfolio

https://doi.org/10.1038/s41590-021-01084-z

Supplementary information

An immunodominant $NP_{105-113}$-B*07:02 cytotoxic T cell response controls viral replication and is associated with less severe COVID-19 disease

In the format provided by the authors and unedited

SUPPLEMENTARY INFORMATION

Supplementary Table 2. Genes used for gene module scoring in single cell expression analysis.

Cytotoxicity	Inhibitory receptors
GZMA	KLRC1
GZMB	PDCD1
GZMH	CD200R1
GZMK	LAG3
PRF1	HAVCR2
GNLY	CD160
NKG7	CTLA4
	BTLA
	TIGIT

Supplementary Table 6. COvid-19 Multi-omics Blood ATlas (COMBAT) Consortium.

David J Ahern, Zhichao Ai, Mark Ainsworth, Chris Allan, Alice Allcock, Azim Ansari, Carolina V Arancibia-Cárcamo, Dominik Aschenbrenner, Moustafa Attar, J. Kenneth Baillie, Eleanor Barnes, Rachael Bashford-Rogers, Archana Bashyal, Sally Beer, Georgina Berridge, Amy Beveridge, Sagida Bibi, Tihana Bicanic, Luke Blackwell, Paul Bowness, Andrew Brent, Andrew Brown, John Broxholme, David Buck, Katie L Burnham, Helen Byrne, Susana Camara, , Philip Charles, Wentao Chen, Yi-Ling Chen, Amanda Chong, Elizabeth Clutterbuck, Mark Coles, Christopher P. Conlon, Richard Cornall, Adam P Cribbs, Fabiola Curion, Emma E Davenport, Neil Davidson, Simon Davis, Calliope Dendrou, Julie Dequaire, Lea Dib, James Docker, Christina Dold, Tao Dong, Damien Downes, Alexander Drakesmith, Susanna J. Dunachie, David A. Duncan, Chris Eijsbouts, Robert Esnouf, Alexis Espinosa, Rachel Etherington, Benjamin Fairfax, Rory Fairhead, Hai Fang, Shayan Fassih, Sally Felle, Maria Fernandez Mendoza, Ricardo Ferreira, Roman Fischer, Thomas Foord, Aden Forrow, John Frater, Anastasia Fries, Veronica Gallardo Sanchez, Lucy Garner, Clementine Geeves, Dominique Georgiou, Leila Godfrey, Tanya Golubchik, Maria Gomez Vazquez, Angie Green, Hong Harper, Heather A Harrington, Raphael Heilig, Svenja Hester, Jennifer Hill, Charles Hinds, Clare Hird, Ling-Pei Ho, Renee Hoekzema, Benjamin Hollis, Jim Hughes, Paula Hutton, Matthew Jackson, Ashwin Jainarayanan, Anna James-Bott, Kathrin Jansen, Katie Jeffery, Elizabeth Jones, Luke Jostins, Georgina Kerr, David Kim, Paul Klenerman, Julian C. Knight, Vinod Kumar, Piyush Kumar Sharma, Prathiba Kurupati, Andrew Kwok, Angela Lee, Aline Linder, Teresa Lockett, Lorne Lonie, Maria Lopopolo, Martyna Lukoseviciute, Jian Luo, Spyridoula Marinou, Brian Marsden, Jose Martinez, Philippa Matthews, Michalina Mazurczyk, Simon McGowan, Stuart McKechnie, Adam Mead, Alexander J Mentzer, Yuxin Mi, Claudia Monaco, Ruddy Montadon, Giorgio Napolitani, Isar Nassiri, Alex Novak, Darragh O'Brien, Daniel O'Connor, Denise O'Donnell, Graham Ogg, Lauren Overend, Inhye Park, Ian Pavord, Yanchun Peng, Frank Penkava, Mariana Pereira Pinho, Elena Perez, Andrew J Pollard, Fiona Powrie, Bethan Psaila, T. Phuong Quan, Emmanouela Repapi, Santiago Revale, Laura Silva-Reyes, Jean-Baptiste Richard, Charlotte Rich-Griffin, Thomas Ritter, Christine S Rollier, Matthew Rowland, Fabian Ruehle,

Mariolina Salio, Stephen Nicholas Sansom, Alberto Santos Delgado, Tatjana Sauka-Spengler, Ron Schwessinger, Giuseppe Scozzafava, Gavin Screaton, Anna Seigal, Malcolm G Semple, Martin Sergeant, Christina Simoglou Karali, David Sims, Donal Skelly, Hubert Slawinski, Alberto Sobrinodiaz, Nikolaos Sousos, Lizzie Stafford, Lisa Stockdale, Marie Strickland, Otto Sumray, Bo Sun, Chelsea Taylor, Stephen Taylor, Adan Taylor, Supat Thongjuea, Hannah Thraves, John A Todd, Adriana Tomic, Orion Tong, Amy Trebes, Dominik Trzupek, Felicia Anna Tucci, Lance Turtle, Irina Udalova, Holm Uhlig, Erinke van Grinsven, Iolanda Vendrell, Marije Verheul, Alexandru Voda, Guanlin Wang, Lihui Wang, Dapeng Wang, Peter Watkinson, Robert Watson, Michael Weinberger, Justin Whalley, Lorna Witty, Katherine Wray, Luzheng Xue, Hing Yuen Yeung, Zixi Yin, Rebecca K Young, Jonathan Youngs, Ping Zhang, Yasemin-Xiomara Zurke
