# Supplementary material for: An immunodominant NP105–113-B*07:02 cytotoxic T cell response controls viral replication and is associated with less severe COVID-19 disease
Source: Nat Immunol. 2021 Dec 1;23(1):50–61. doi: 10.1038/s41590-021-01084-z (PMC8709787; doi:10.1038/s41590-021-01084-z)
Supplement: Supplementary file 2 — Reporting Summary [file 41590_2021_1084_MOESM2_ESM.pdf]

nature portfolio

Corresponding author(s): Tao Dong

Last updated by author(s): Oct 14, 2021

Reporting Summary

Nature Portfolio wishes to improve the reproducibility of the work that we publish. This form provides structure for consistency and transparency in reporting. For further information on Nature Portfolio policies, see our Editorial Policies and the Editorial Policy Checklist.

Statistics

For all statistical analyses, confirm that the following items are present in the figure legend, table legend, main text, or Methods section.

n/a	Confirmed	
☐	☒	The exact sample size (*n*) for each experimental group/condition, given as a discrete number and unit of measurement
☐	☒	A statement on whether measurements were taken from distinct samples or whether the same sample was measured repeatedly
☐	☒	The statistical test(s) used AND whether they are one- or two-sided<br>*Only common tests should be described solely by name; describe more complex techniques in the Methods section.*
☐	☒	A description of all covariates tested
☐	☒	A description of any assumptions or corrections, such as tests of normality and adjustment for multiple comparisons
☐	☒	A full description of the statistical parameters including central tendency (e.g. means) or other basic estimates (e.g. regression coefficient) AND variation (e.g. standard deviation) or associated estimates of uncertainty (e.g. confidence intervals)
☐	☒	For null hypothesis testing, the test statistic (e.g. *F*, *t*, *r*) with confidence intervals, effect sizes, degrees of freedom and *P* value noted<br>*Give P values as exact values whenever suitable.*
☒	☐	For Bayesian analysis, information on the choice of priors and Markov chain Monte Carlo settings
☒	☐	For hierarchical and complex designs, identification of the appropriate level for tests and full reporting of outcomes
☒	☐	Estimates of effect sizes (e.g. Cohen's *d*, Pearson's *r*), indicating how they were calculated

Our web collection on statistics for biologists contains articles on many of the points above.

Software and code

Policy information about availability of computer code

Data collection	ELISpot data were collected with AID ELISpot 7.0 and flow cytometry data were collected by BD FACSDiva V9.0/ Attune™ NxT software V3.2.1 Sequencing data were collected with NextSeq Control Software (NCS) version 4 or MiSeq Control Software Version 2.6.2.1
Data analysis	Flow cytometry data were analyzed with FlowJoTM v.10.5.3 software for Mac. Prism version 8.2.1 for Mac OS. Raw BCL files from SmartSeq2 single cell RNA sequencing were converted to FASTQ format using bcl2fastq (v 2.20.0.422). FASTQ files were aligned to human genome hg19 using STAR (v 2.6.1D). Reads were counted using featureCounts (part of subread v 2.0.0). Resulting count matrices were analysed in R v 4.0.1 using Seurat (v 3.9.9.9010). Raw BCL files from 10x sequencing were processed using 10x Genomics Cellranger (v 5.0.0). Genetic demultiplexing was conducted using cellSNP (v 0.3.2) and Vireo (v 0.5.6). Packages used in R for additional analyses or data visualisation are: Harmony (v 1.0), ggplot2 (v 3.3.2), tcR (v 2.3.2), circlize (v 0.4.12), stringdist (v 0.9.6), ggalluvial (v 0.12.2), DESeq2 (v 1.28.1), pheatmap (v 1.0.12). TCR sequences were reconstructed from FASTQ files using MiXCR (v 3.0.13). GLIPH2 irtools.centos (v 0.01) run on a CentOS Linux platform (release 8.2.2011) was used for TCR analysis.

For manuscripts utilizing custom algorithms or software that are central to the research but not yet described in published literature, software must be made available to editors and reviewers. We strongly encourage code deposition in a community repository (e.g. GitHub). See the Nature Portfolio guidelines for submitting code & software for further information.

Data

Policy information about availability of data

All manuscripts must include a data availability statement. This statement should provide the following information, where applicable:

- Accession codes, unique identifiers, or web links for publicly available datasets
- A description of any restrictions on data availability
- For clinical datasets or third party data, please ensure that the statement adheres to our policy

The raw data from all the main figures and supplementary figures are available upon request. In addition the following published datasets were used: Lineburg et al (https://doi.org/10.1016/j.immuni.2021.04.006), Nguyen et al (https://doi.org/10.1016/j.immuni.2021.04.009) and COMBAT (https://doi.org/10.1101/2021.05.11.21256877)

Single cell raw data (excluding TCR sequences) can only be shared on request due to ongoing work for future publications.

Field-specific reporting

Please select the one below that is the best fit for your research. If you are not sure, read the appropriate sections before making your selection.

☒ Life sciences ☐ Behavioural & social sciences ☐ Ecological, evolutionary & environmental sciences

For a reference copy of the document with all sections, see nature.com/documents/nr-reporting-summary-flat.pdf

Life sciences study design

All studies must disclose on these points even when the disclosure is negative.

Sample size	52 subjects recovered from COVID-19 samples (30 mild and 22 severe). This was a follow-up for a published study (Peng et al, NI, https://doi.org/10.1038/s41590-020-0782-6). Samples sizes were based on maximal available samples sets where detailed clinical and serological data were also available, and aligned well with our previously published data in other settings, including influenza and HIV (Lee et al, JCI 2008; Zhang et al, NC 2013; Zhao et al, AJCCM 2012)
Data exclusions	For ELISPOT assays, If negative control wells had >30 SFU/106 PBMCs or positive control wells (PHA stimulation) were negative, the results were excluded from further analysis. There were no data excluded from ELISpot analysis.<br>For scRNA-Seq analysis cells were filtered using the following criteria: minimum number of cells expressing specific gene = 3, minimum number of genes expressed by cell = 200 and maximum number of genes expressed by cell = 4000. Cells were also excluded if they expressed more than 5% mitochondrial genes.<br>For paired αβ chain TCR analysis, cells were filtered to retain only 1α1β or 2α1β cells.<br>For single β chain TCR analysis, cells were filtered to retain only 1β (regardless of number of α) to use for downstream analysis.
Replication	Samples analyzed in this study were from participants of a cohort study and samples were analyzed on individual study participants. Ex vivo experiments did not include replicates, but results from each participant were confirmed by at least two different experiments.<br>For in vitro assays, results shown are always two or more independent experiments in which every repeat gave similar results.
Randomization	Randomization was not appropriate for this study of immune responses in COVID-19 convalescent individuals, with no associated therapeutic intervention
Blinding	Blinding was not appropriate for this study of immune responses in COVID-19 convalescent individuals, with no associated therapeutic intervention

Reporting for specific materials, systems and methods

We require information from authors about some types of materials, experimental systems and methods used in many studies. Here, indicate whether each material, system or method listed is relevant to your study. If you are not sure if a list item applies to your research, read the appropriate section before selecting a response.

Materials & experimental systems

n/a	Involved in the study	
☐	☒	Antibodies
☐	☒	Eukaryotic cell lines
☒	☐	Palaeontology and archaeology
☒	☐	Animals and other organisms
☐	☒	Human research participants
☒	☐	Clinical data
☒	☐	Dual use research of concern

Methods

n/a	Involved in the study	
☒	☐	ChIP-seq
☐	☒	Flow cytometry
☒	☐	MRI-based neuroimaging

Antibodies

Antibodies used

Antibodies used for flow cytometry

Marker	Fluorophore	Supplier	Cat number	Clonotype	Lot Number	Dilution
CD14	BV510	BioLegend	367124	63D3	B280762	1:50
CD16	BV510	BioLegend	302048	3G8	B289732	1:50
CD19	BV510	BioLegend	302242	HIB19	B281769	1:50
CD8	PerCP-Cy5.5	BD Bioscience	565310	SK1	9312532	1:33
CD8	BV421	BioLegend	344748	SK1	B331004	1:40
CD3	FITC	BD Bioscience	345763	SK7	0342661	1:25
IFNg	AF488	BD Bioscience	557718	B27	9128859	1:33
TNFa	APC	eBioscience	17-7349-82	MAb11	2101117	1:500
MIP1ß	APC-H7	BD Bioscience	561280	D21-1351	1046672	1:33
CD107a	PE	BD Bioscience	555801	H4A3	0073840	1:20
ACE2	Primary	R&D	AF933	Polyclonal	HOK0620051	1:20
anti-goat	AF647	AbCam	ab150135		GR3324428-3	1:1000

Validation

All antibodies used in this study are commercially available. Antibodies used in a specific species or application have been appropriately validated by manufacturers for that application and this information is provided on their website and product information datasheets. All antibodies described here have been further optimized for an appropriate concentration by testing several dilutions.
CD14 https://www.biolegend.com/en-us/products/brilliant-violet-510-anti-human-cd14-antibody-14983
CD16 https://www.biolegend.com/en-us/products/brilliant-violet-510-anti-human-cd16-antibody-8003
CD19 https://www.biolegend.com/en-us/products/brilliant-violet-510-anti-human-cd19-antibody-8004
TNFa https://www.thermofisher.com/antibody/product/TNF-alpha-Antibody-clone-MAb11-Monoclonal/17-7349-82
CD107a https://www.bdbiosciences.com/eu/applications/research/intracellular-flow/intracellular-antibodies-and-isotype-controls/anti-human-antibodies/pe-mouse-anti-human-cd107a-h4a3/p/555801
CD3 https://www.bdbiosciences.com/sg/reagents/clinical/reagents/single-antibodies/cd3-fitc-sk7-also-known-as-leu-4/p/345763
CD8 https://www.bdbiosciences.com/sg/reagents/research/antibodies-buffers/immunology-reagents/anti-non-human-primate-antibodies/cell-surface-antigens/percp-cy55-mouse-anti-human-cd8-sk1/p/565310
MIP1β https://www.bdbiosciences.com/sg/applications/research/intracellular-flow/intracellular-antibodies-and-isotype-controls/anti-human-antibodies/apc-h7-mouse-anti-human-mip-1-d21-1351/p/561280
IFNγ https://www.bdbiosciences.com/sg/applications/research/t-cell-immunology/th-1-cells/intracellular-markers/cytokines-and-chemokines/human/alexa-fluor-488-mouse-anti-human-ifn--b27/p/557718
CD8 https://www.biolegend.com/en-gb/products/brilliant-violet-421-anti-human-cd8-antibody-13512
ACE2 https://www.rndsystems.com/products/human-mouse-rat-hamster-ace-2-antibody_af933
donkey anti-goat AF647 https://www.abcam.com/donkey-goat-igg-hl-alexa-fluor-647-preadsorbed-ab150135.html

Eukaryotic cell lines

Policy information about cell lines

Cell line source(s)

All the EBV-transformed B cell lines were established in the lab. Vero E6 cells (ATCC CCL-81), HEK293T cells (ATCC, CRL-11268), TK143B cells (ATCC, CRL-8303), rabbit RK13 cells (ATCC, CCL37) and African green monkey BS-C-1 cells (ATCC, CCL26) were from ATCC. Vero E6/TMPRSS2 cells were originally from NIBSC (reference 100978).

Authentication

Cell lines were validated by morphology. Vero E6 cells, HEK293T cells, TK143B cells, rabbit RK13 cells and BS-C-1 cells were obtained from ATCC and used at low passage. They were not further authenticated. Vero E6/TMPRSS2 from NIBSC were used for propagating SARS-CoV-2 virus and were not further authenticated. EBV transformed B cell lines expressing ACE2 were verified by flow cytometry staining of ACE2 and CD19.

Mycoplasma contamination

All the cell lines were tested negative for mycoplasma

Commonly misidentified lines
(See ICLAC register)

No misidentified cell lines were used according to the version 11 of register of misidentified cell lines

Human research participants

Policy information about studies involving human research participants

Population characteristics

52 individuals were recruited following recovery from COVID-19, including 30 mild cases and 22 severe cases. Supplementary Figure 1 shows the participant characteristics. No significant differences in gender or age were noted between mild and severe groups, which was shown in previous publication (https://doi.org/10.1038/s41590-020-0782-6). The SaO2/FiO2 ratio in severe cases ranged from 4.3 (where 4.5 would be the estimate for an individual with mild disease breathing ambient air) to 1.6 with the patients with critical disease having an estimate of 0.8 (median in severe group 3.8).

Recruitment

Patients were recruited from the John Radcliffe Hospital in Oxford, UK, between March 2020 and April 2021 by identification of patients hospitalised during the SARS-COV-2 pandemic. Patients were recruited into the Sepsis Immunomics study and had samples collected during their convalescence as well as during acute disease. For convalescence, patients were sampled at least 28 days from the start of their symptoms. Written informed consent was obtained from all patients. The potential bias, such as the timing when samples were taken, the gender and age of the patients, are unlikely to impact the results, as there is no significant difference in the age between two study groups, and no correlation was observed between the T cell

response and days post symptoms when samples were taken, as shown in previous publication (https://doi.org/10.1038/s41590-020-0782-6)

Ethics oversight

The samples were collected from patients with confirmed COVID who had consented to participate in Sepsis Immunomic project IRAS 260007. Ethical approval was given by the South Central-Oxford C Research Ethics Committee in England (Ref 19/SC/0296).

Note that full information on the approval of the study protocol must also be provided in the manuscript.

Flow Cytometry

Plots

Confirm that:

☒ The axis labels state the marker and fluorochrome used (e.g. CD4-FITC).

☒ The axis scales are clearly visible. Include numbers along axes only for bottom left plot of group (a 'group' is an analysis of identical markers).

☒ All plots are contour plots with outliers or pseudocolor plots.

☒ A numerical value for number of cells or percentage (with statistics) is provided.

Methodology

Sample preparation

Cryopreserved PBMCs were thawed and rested overnight in R10 at 37oC. On the second day, PBMCs were stained with PE-conjugated HLA-B7 NP105-113 Pentamer (ProImmune, Oxford, UK). Live/Dead fixable Aqua dye (Invitrogen) was used to exclude non-viable cells from the analysis. Subsequently, cells were washed and stained with the following surface antibodies: CD3-FITC (BD Biosciences), CD8-PercP-Cy5.5, CD14-BV510, CD19-BV510 and CD16-BV510 (Biolegend). After the final wash, cells were resuspended in 500µl of PBS, 2 mM EDTA and 0.5% BSA (Sigma-Aldrich) solution and kept in dark at 4°C until flow cytometric acquisition. After exclusion of non-viable/CD19+/CD14+/CD16+ cells, CD3+ CD8+ Pentamer+ cells were sorted directly into 96-well PCR plates containing cell lysis buffer using a BD Fusion 1 or BD FACS Aria III (BD Biosciences). For intracellular cytokine staining (ICS), in vitro expanded T cell lines/clones were co-cultured with autologous B cell lines loaded with peptides, or infected with Vacinnia virus/live virus at an appropriate E:T ratio, in the presence of GolgiPlug (brefeldin A, BD), GolgiStop (monensin, BD) and 5ul of PE-anti-CD107a (BD Biosciences) for 5hrs. Then a standard FACS staining was carried out. Briefly, dead cells were first labelled with LIVE/DEAD™ Fixable Aqua dye and then followed by surface antibody staining. Subsequently, Cytofix/CytopermTM kit (BD Biosciences) was used for permeabillizing the cells before staining the cells with antibodies against molecules expressed intracellularly. Finally, cells then be fixed with 1X cell fixing buffer.

Instrument

Samples were sorted using BD Fusion 1 or BD FACS Aria III (BD Biosciences) or acquired at Thermo Fisher Attune™ NxT Flow Cytometer

Software

Data were collected using FACS DIVA (v9.0.1) or Attune™ NxTsoftware v3.2.1 and analyzed using FlowJoTM v.10 software for Mac.

Cell population abundance

All sorted samples were checked for after-sorting purity (>99%)

Gating strategy

For all the experiments, cells were first gated on single Lymphocytes by a forward side scatter gate. On sorting for RNASeq, after excluding dead cells, CD14+, CD19+, and CD16+ cells, HLA-B7-NP 105-113 specific T cells were identified as CD3+CD8+Pentamer+. For intracellular cytokine staining (ICS), the cytokine positive/ negative population were gated according to corresponding negative controls, known as unstimulated samples (T cells co-cultured with target cells without peptide loading or without virus infection): after excluding dead cells, cells then were gated into CD8+ T cells IFN-g+/-, TNFa+/-, MIP11ß+/- and CD107a+/- populations were gated in consistence with the corresponding negative controls.

☒ Tick this box to confirm that a figure exemplifying the gating strategy is provided in the Supplementary Information.
