# Supplementary material for: An immunodominant NP105–113-B*07:02 cytotoxic T cell response controls viral replication and is associated with less severe COVID-19 disease
Source: Nat Immunol. 2021 Dec 1;23(1):50–61. doi: 10.1038/s41590-021-01084-z (PMC8709787; doi:10.1038/s41590-021-01084-z)
Supplement: Supplementary file 3 — Peer Review Information [file 41590_2021_1084_MOESM3_ESM.pdf]

natureresearch

Peer Review Information

Journal: Nature Immunology
Manuscript Title: An immunodominant NP105-113-B*07:02 cytotoxic T cell response controls viral replication and is associated with less severe COVID-19 disease
Corresponding author name(s): Tao Dong

Reviewer Comments & Decisions:

Decision Letter, initial version:

17th Aug 2021

Dear Professor Dong,

Your Article, "Immunodominant NP105-113-B*0702 cytotoxic T cell response controls viral replication and is associated with less severe COVID-19 disease" has now been seen by 2 referees. You will see from their comments below that while they find your work of interest, some important points are raised. We are very interested in the possibility of publishing your study in Nature Immunology, but would like to consider your response to these concerns in the form of a revised manuscript before we make a final decision on publication.

We therefore invite you to revise your manuscript taking into account all reviewer and editor comments. Please highlight all changes in the manuscript text file in Microsoft Word format.

We are committed to providing a fair and constructive peer-review process. Do not hesitate to contact us if there are specific requests from the reviewers that you believe are technically impossible or unlikely to yield a meaningful outcome.

When revising your manuscript:

* Include a “Response to referees” document detailing, point-by-point, how you addressed each referee comment. If no action was taken to address a point, you must provide a compelling argument. This response will be sent back to the referees along with the revised manuscript.

* If you have not done so already please begin to revise your manuscript so that it conforms to our Article format instructions at http://www.nature.com/ni/authors/index.html. Refer also to any guidelines provided in this letter.

* Please include a revised version of any required reporting checklist. It will be available to referees to aid in their evaluation of the manuscript goes back for peer review. They are available here:

Reporting summary:
https://www.nature.com/documents/nr-reporting-summary.pdf

When submitting the revised version of your manuscript, please pay close attention to our href="https://www.nature.com/nature-research/editorial-policies/image-integrity">Digital Image Integrity Guidelines. and to the following points below:

-- that unprocessed scans are clearly labelled and match the gels and western blots presented in figures.
-- that control panels for gels and western blots are appropriately described as loading on sample processing controls
-- all images in the paper are checked for duplication of panels and for splicing of gel lanes.

Finally, please ensure that you retain unprocessed data and metadata files after publication, ideally archiving data in perpetuity, as these may be requested during the peer review and production process or after publication if any issues arise.

Please use the link below to submit your revised manuscript and related files: [REDACTED]

Note: This URL links to your confidential home page and associated information about manuscripts you may have submitted, or that you are reviewing for us. If you wish to forward this email to co-authors, please delete the link to your homepage.

We hope to receive your revised manuscript within four weeks. If you cannot send it within this time, please let us know. We will be happy to consider your revision so long as nothing similar has been accepted for publication at Nature Immunology or published elsewhere.

Please do not hesitate to contact me if you have any questions or would like to discuss these revisions further.

Nature Immunology is committed to improving transparency in authorship. As part of our efforts in this direction, we are now requesting that all authors identified as ‘corresponding author’ on published papers create and link their Open Researcher and Contributor Identifier (ORCID) with their account on the Manuscript Tracking System (MTS), prior to acceptance. ORCID helps the scientific community achieve unambiguous attribution of all scholarly contributions. You can create and link your ORCID from the home page of the MTS by clicking on ‘Modify my Springer Nature account’. For more information please visit please visit www.springernature.com/orcid.

We look forward to seeing the revised manuscript and thank you for the opportunity to review your work.

Sincerely,

Zoltan Fehervari, Ph.D.
Senior Editor
Nature Immunology

The Macmillan Building
4 Crinan Street
Tel: 212-726-9207
Fax: 212-696-9752
z.fehervari@nature.com

Referee expertise:

Referee #1: T cells, Infectious disease

Referee #2: T cells, Infectious disease

Reviewers' Comments:

Reviewer #1:
Remarks to the Author:
This manuscript entitled, “An immunodominant NP-B*07:02 cytotoxic T cell response controls viral replication and is associated with less severe COVID-19 disease”, describes results analyzing NP-specific

CD8 T cells in a small cohort of patients who recovered from mild or severe COVID-19, with in-depth, single cell analysis of NP-reactive CD8 T cells from 2 patients who recovered from mild and 2 from severe disease comprising the majority of the results presented. The authors and others have previously identified NP105-113 as an immunodominant epitope presented by individuals with HLA-B*07:02 alleles. They find slightly higher frequencies of NP-reactive CD8 T cells from individuals with mild (n=10) compared to severe (n=9) disease. By scRNAseq, they identified more cytotoxicity in T cells from severe disease, and using existing datasets found a higher similarity score for NP-reactive T cells from individuals who recovered from mild disease, compared to more high avidity T cells in individuals who recovered from mild disease, while both low and high avidity T cells showed anti-viral activity in an in vitro cytotoxicity assay on SARS-CoV2-infected target cells. Overall, the analysis is interesting, and the single cell+TCR dataset is valuable to the field. Following the logic of the analysis was confusing in certain figures where the analysis of the scRNAseq data was mixed in with existing datasets, or compared to subsets of existing datasets. The major conclusions derived concerning the association of specific clonotypes or functional avidity to disease severity are less convincing and other possibilities could be considered (see itemized comments below).

1 There is a paucity of information on the individuals being studied. It would be helpful to include a table of the overall characteristics of the cohorts being studied—stratified by mild and severe, including information on age range, overall severity, time post symptom onset, and p values on whether they are different between the groups. Maybe this information is elsewhere, but I couldn't find the precise references to it in the manuscript, and in any case, it is important to link the subject information to the study. It would be helpful to provide the age of all the donors for which TCR data is obtained and analyzed, as age can impact TCR diversity, subset delineation, and clonal expansion. Older individuals have decreased TCR diversity, increased clonal expansion, increased TEMRA cells, and other functional alterations—which are particularly manifested among CD8 T cells.

2. The authors perform simultaneous scRNAseq +TCR analysis for the NP reactive T cells, but most of the analysis is showing a very limited subset of the data. For example, for the gene expression results, the only data shown are the compiled expression data for a very limited subset of genes (cytotoxicity and inhibitory) that differ between mild and severe, and one does not get a sense of the overall T cell gene expression found for these NP-reactive cells. Can the authors present tSNE or UMAPs of the gene expression data combined from all 4 donors, to show whether or not the cells segregate by individual or disease severity, using unbiased clustering. With this UMAP, it would also be possible to show specific clones and in what cluster they are found. For example, are the highly expanded clones identified found mostly in the TEMRA cells—were those cells more cytotoxic (or less?). Were there more or fewer highly expanded clones in the severe or mild group?

3. The comparison on the TCR results to existing datasets in Fig. 4 is difficult to interpret. For Fig. 4A, the authors compare the similarity of the TCRs from the 2 mild and 2 severe patients to TCR from healthy

donors. The box and whisker plots overlap but this statistical analysis indicates significance. Were the same number of TCRs compared for milda nd severe groups because the legend indicates 85 clonotypes, but not hpw many for each. For Figure 4B, additional data was used in the comparisons—including 738 TCRs from 12 mild patients (Does this data include the data from the 4 patients analyzed here? For the data in Figure 4B, a higher number of TCRs are compared for mild patients, than for severe (735 TCRs versus 133TCRs). Won't this discrepancy affect the results?—i.e., if you are comparing more TCRs you will have a greater chance of finding similar clones. Accordingly, the reduced number of similar clones in the severe relative to the mild groups is because nearly 6-fold fewer clones were analyzed. Related to this point, the fewer TCR clones identified in the servere group could be due to age—although age of the individuals studied was not provided (see comment #1 above), it is well established that individuals who suffer from severe COVID-19 tend to be older. The reduced TCR diversity (and reduced similarity to healthy controls) could be due to reduced TCR diversity overall in older individuals.

4. The TCR avidity results are interesting, and the assay of T cell killing on virally-infected T cells provides a robust assessment of potential protective capacity. However, using this assay to derive firm conclusions on whether patients who recovered from mild disease maintain more high- avidity clones requires a higher number of individuals. For example, in Fig. 7, one of the individuals who recovered from mild disease maintained both high and low avidity clones similar to the proportions seen in the severe patients analyzed by scRNAseq (Fig. 6).

Reviewer #2:

Remarks to the Author:

Peng and co-workers performed an in-depth analysis of SARS-CoV-2 specific CD8+ T cells targeting the HLA-B*07:02-restricted NP105-113 epitope that has been described to be immunodominant by several groups. While this epitope-specific CD8+ T cell response has already been specifically studied in two previous papers published in IMMUNITY, the current manuscript clearly further extends the previous findings by adding important novel aspects. First, it links the strength of the response (but not presence of HLA-B*07:02 per se) to a mild course of infection. Second, they demonstrate that epitope-specific CD8+ T cells in patients with severe course display higher cytotoxicity and inhibitory receptor expression compared to patients with mild course. Third, TCRs from patients with mild course have higher similarity to TCRs from naïve precursors found in pre-pandemic samples and display a higher avidity. Fourth, the relevance of higher avidity TCRs is confirmed in natural processing as well as in vitro infection experiments. Finally, a proportionally narrowing of the TCR repertoire six months after infection comes along with maintenance of antiviral efficacy against prototype as well as variant (alpha, beta, gamma) virus. Thus, this study gives important insights into protective CD8+ T cell immunity in natural SARS-CoV-2 infection.

There are, however, also some issues that need additional attention.

1. The authors state e.g. in the abstract “strong association of this response with mild disease”. It is extremely important, however, to clearly state throughout the manuscript that the strength of the response, but not the presence of the response per se, is associated with mild disease. In this context, it is important to point out that Fig. 1b is extremely misleading/ incorrect. This panel refers to the 15 B*07:02+ patients who respond to the epitope. 9 of these (60%) had mild disease, 6 (40%) severe disease. This distribution is completely the same as the distribution in the overall cohort (30 mild cases; 22 severe cases). Thus, responding to the epitope per se is not associated with mild infection. The data displayed in Fig. 1B thus (1) should be displayed as a pie chart and (2) needs to be compared to the overall cohort.
2. The authors argue that higher cytotoxicity gene expression scores as well as increased inhibitory receptor expression in patients with severe infection argue for exposure to higher antigen load, meaning that they are a consequence rather than a cause of severe infection. The same may be due to TCR similarity/divergence from naïve precursors as well as avidity, e.g. these may be a consequence rather than cause for mild versus severe disease. This limitation should be discussed more carefully.
3. Fig. 7D: VOC alpha, beta, and gamma are studied here, but delta is missing. Since delta is the most relevant VOC at presence, it should also be analyzed here.
Minor:
1. HLA-B*07:02 is incorrectly spelled as HLA-B*0702 at multiple sites of the manuscript.
2. Line 164: “…by other studies (ref 7)..”: Are there additional studies to ref 7?
3. Line 179: “Both studies…”: There are four references – please clarify.
4. Lines 182-184: “Overall, our data supported the notion… (ref 7)”: This sentence is unclear to me. Unexposed individuals have not been studied here, and ref 7 does not contain data by the authors of this study.
5. Discussion, lines 312-313: “We found high similarity of TCR in both HLA-B*07:02 positive and HLA-B*07:02 negative COVID-19 recovered individuals…”: What does this “similarity” refer to? The sentence is not clear to me.
6. Discussion, lines 359-362: “…high frequency naïve T cell precursors recognizing NP105-113-B*07:02 that are observed in pre-pandemic individuals are HLA-B*07:02 independent…”: Where is this shown? Are they also present in HLA-B*07:02 negative individuals? This is not clear to me.

Author Rebuttal to Initial comments

Dear Dr. Fehervari:

We greatly appreciate the opportunity to revise our manuscript for publication within Nature Immunology and address the concerns raised by the reviewers. We have now revised our manuscript, and highlighted all changes. The detailed responses follow, but for ease of reference we have made the following changes:

1. As requested, the manuscript has been carefully clarified throughout.
2. New Figure 1B, Supplementary Table 1 and Supplementary Figure 1 were added as requested by the reviewers
3. New data generated since the submission have now been included in the revised manuscript as:
   - New intracellular cytokine data on bulk T cell lines from month 6 post COVID recovery in recognition of five Variants of Concern including the newly emerged Delta variant currently circulating in UK (Figure 7B and Figure S5)
   - Virus suppression assay with Delta variant required by reviewer 2 (Figure 7E)

4. Additional revisions including supplementary figures and tables
   - We have added three new co-authors Chang Liu, Prathiba Kurupati and Bo Sun in recognition of their contribution to this work.
   - We have updated the numbering for the supplementary figures and tables due to the addition of new data:
     - Fig. S1. Participant characteristics.
     - Fig. S2. TCR clonotypes for single cells and T cell clones and EC50 derivation.
     - Fig. S3. FACS gating strategy and peptide titration.
     - Fig. S4. T cell response six months post infection to NP Vaccinia virus.
     - Fig. S5. T cell response six months post infection to SARS-CoV-2 and VOCs
     - Table S1. Participant characteristics
     - Table S2. Genes used for gene module scoring in single cell expression analysis.
     - Table S3. αβ VJ gene usage and CDR3 sequences for ex vivo single cell TCR sequencing.
     - Table S4. αβ VJ gene usage and CDR3 sequences from bulk TCR sequencing of T cell clones.
     - Table S5. EC50 values from selected T cell clones.
     - Table S6. COvid-19 Multi-omics Blood ATlas (COMBAT) Consortium.

5. We have formatted the paper according to Nature Immunology guidelines.

The following are the **point by point responses to reviewers' comments:**

Reviewer #1:

C: Remarks to the Author:

This manuscript entitled, “An immunodominant NP-B*07:02 cytotoxic T cell response controls viral replication and is associated with less severe COVID-19 disease”, describes results analyzing NP-specific CD8 T cells in a small cohort of patients who recovered from mild or severe COVID-19, with in-depth, single cell analysis of NP-reactive CD8 T cells from 2 patients who recovered from mild and 2 from severe disease comprising the majority of the results presented. The authors and others have previously identified NP105-113 as an immunodominant epitope presented by individuals with HLA-B*07:02 alleles. They find slightly higher frequencies of NP-reactive CD8 T cells from individuals with mild (n=10) compared to severe (n=9) disease. By scRNAseq, they identified more cytotoxicity in T cells from severe disease, and using existing datasets found a higher similarity score for NP-reactive T cells from individuals who recovered from mild disease, compared to more high avidity T cells in individuals who recovered from mild disease, while both low and high avidity T cells showed anti-viral activity in an in vitro cytotoxicity assay on SARS-CoV2-infected target cells. Overall, the analysis is interesting, and the single cell+TCR dataset is valuable to the field. Following the logic of the analysis was confusing in certain figures where the analysis of the scRNAseq data was mixed in with existing datasets, or compared to subsets of existing datasets. The major conclusions derived concerning the association of specific clonotypes or functional avidity to disease severity are less convincing and other possibilities could be considered (see itemized comments below).

R: We appreciate the positive comments from this reviewer.

Specific Comments:

C1: There is a paucity of information on the individuals being studied. It would be helpful to include a table of the overall characteristics of the cohorts being studied—stratified by mild and severe, including information on age range, overall severity, time post symptom onset, and p values on whether they are different between the groups. Maybe this information is elsewhere, but I couldn’t find the precise references to it in the manuscript, and in any case, it is important to link the subject information to the study. It would be helpful to provide the age of all the donors for which TCR data is obtained and analyzed, as age can impact TCR diversity, subset delineation, and clonal expansion. Older individuals have decreased TCR diversity, increased clonal expansion, increased TEMRA cells, and other functional alterations—which are particularly manifested among CD8 T cells.

R1: We have added a new Supplementary Figure 1 with the overall characteristics of the cohorts being studied, stratified by mild and severe, and including information on age range, overall severity, time post symptom onset, and *P* values on whether they are statistically different between severity groups. We have a new Supplementary Table 1 which lists the age and severity

(where appropriate) of COVID-19 patients in our cohort, healthy/pre-pandemic individuals (from Oxford COMBAT, Nguyen and Lineburg cohorts) and Oxford COMBAT COVID-19 patients. We have also included the age of donors for SmartSeq2 RNA-Seq in the appropriate Results section.

Results, page 4: "clinical features summarised in Supplementary Table 1 and Figure S1A-C"

Results, page 4: "(C-COV19-005, 56 years old and C-COV19-046, 76 years old) and two who recovered from severe disease in early infection (C-COV19-038, 44 years old and C-COV19-045, 72 years old)"

Figure S1: "**Participant characteristics.** (A) Distribution of age, gender and days post symptom onset when sampling of SARS-CoV-2 infected patients. (B) and (C) Comparison of age (ns, *P*=0.116) and days post symptom (ns, *P*=0.5711) when sampling between the patient groups with mild and severe symptoms. Unpaired t-test with Welch's correction and Mann-Whitney test were used for data analysis of (B) and (C) respectively. Two tailed *P* value was calculated. (D) Breakdown of patient numbers and severity; HLA-B*07:02 negative (mild N=20 light grey, N=13 dark grey); HLA-B*07:02 positive responders (mild N=9 red, severe N=6 blue); HLA-B*07:02 positive non-responders (mild N=1 light red, severe N=3 light blue)."

Table S1: "**Participant characteristics.** Gender, days post symptom onset and age shown for each participant."

C2: The authors perform simultaneous scRNAseq +TCR analysis for the NP reactive T cells, but most of the analysis is showing a very limited subset of the data. For example, for the gene expression results, the only data shown are the compiled expression data for a very limited subset of genes (cytotoxicity and inhibitory) that differ between mild and severe, and one does not get a sense of the overall T cell gene expression found for these NP-reactive cells. Can the authors present tSNE or UMAPs of the gene expression data combined from all 4 donors, to show whether or not the cells segregate by individual or disease severity, using unbiased clustering. With this UMAP, it would also be possible to show specific clones and in what cluster they are found. For example, are the highly expanded clones identified found mostly in the TEMRA cells—were those cells more cytotoxic (or less?). Were there more or fewer highly expanded clones in the severe or mild group?

R2: Analysis of total CD8+ NP-specific sorted T cells by single cell RNA-Seq with UMAP visualisation and unbiased clustering revealed a homogenous population (see UMAP below – labelled by patient ID, Seurat clusters and severity). There are very few significantly differentially expressed genes between cluster 0 and 1, likely reflecting the limitations of the cluster based approach in the setting of a small number of total CD8+ NP-specific sorted T cells. Therefore, we believe it was more useful to focus on specific gene sets instead of gene expression by clustering. We have added a sentence to the Results section to justify our focus on expression of gene modules. We appreciate it would be interesting to look gene signatures associated with highly expanded clonotypes, however, we did not find any unique highly expanded clonotypes; instead, as shown in Figure 2, we observed highly diverse TCR repertoires.

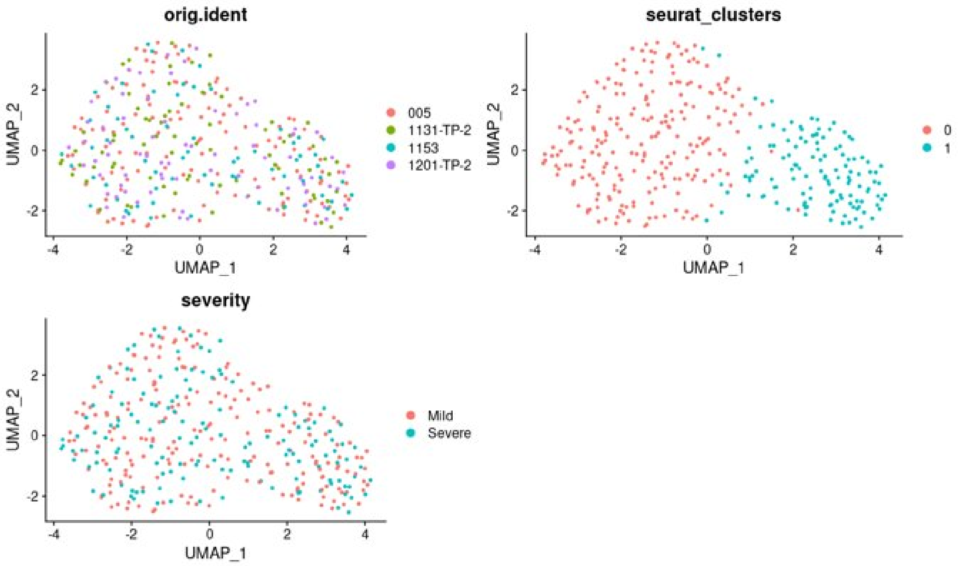

Results page 4: "Analysis of single cell RNA-Seq data with UMAP visualisation and unbiased clustering revealed a homogenous cell population; therefore we compared gene expression of CD8^{+} NP-specific sorted single cells isolated from mild (N=208 from two patients) and severe cases (N=140 from two patients) by scoring expression levels of manually defined gene sets (Supplementary Table 2)."

C3: The comparison on the TCR results to existing datasets in Fig. 4 is difficult to interpret.

C3.1: For Fig. 4A, the authors compare the similarity of the TCRs from the 2 mild and 2 severe patients to TCR from healthy donors. The box and whisker plots overlap but this statistical analysis indicates significance. Were the same number of TCRs compared for mild and severe groups because the legend indicates 85 clonotypes, but not how many for each. For Figure 4B, additional data was used in the comparisons—including 738 TCRs from 12 mild patients (Does this data include the data from the 4 patients analyzed here?

R3.1: We have added additional text in the Results to clarify that Fig 4A and 4B represent two different methodologies to compare NP-specific TCRs from healthy and COVID-19 convalescent individuals. We have added in the figure legend that out of the 85 unique clonotypes, 38 are associated with mild cases and 47 with severe disease. The reviewer is correct that Figure 4B includes the data from the 4 patients analysed, we have now made this clearer in the text.

Results, page 5/6: "We sought to compare NP-specific TCRs from COVID patients and healthy individuals using two different methodologies. Firstly, we calculated similarity scores for CDR3β amino acid sequences between pairwise combinations of SmartSeq2 TCRs and pre-pandemic/healthy TCRs… (Figure 4A, P<2.20E^{-16}). Secondly, we looked at the proportion of TCR sequences from mild and severe patients … (Figure 4B, P<2.2E^{-16})."

Legend, figure 4: "(**A**) Similarity scores from pairwise comparisons between TCRs from pre-pandemic individuals (237 TCRs) and 85 unique clonotypes from convalescent COVID-19 patients (38 mild TCRs v 47 severe TCRs)."

Results, page 6: "Secondly, we looked at the proportion of TCR sequences from mild and severe patients (acute cases from COMBAT dataset and convalescent cases from previously described SmartSeq2 patients) that can be found in the same convergence groups as sequences from healthy donors, indicating high CDR3β similarity."

C3.2: For the data in Figure 4B, a higher number of TCRs are compared for mild patients, than for severe (735 TCRs versus 133TCRs). Won't this discrepancy affect the results?—i.e., if you are comparing more TCRs you will have a greater chance of finding similar clones. Accordingly, the reduced number of similar clones in the severe relative to the mild groups is because nearly 6-fold fewer clones were analyzed. Related to this point, the fewer TCR clones identified in the severe group could be due to age—although age of the individuals studied was not provided (see comment #1 above), it is well established that individuals who suffer from severe COVID-19 tend

to be older. The reduced TCR diversity (and reduced similarity to healthy controls) could be due to reduced TCR diversity overall in older individuals.

R3.2: While we agree this difference in Figure 4B might affect the result, the data from Fig 4A (from more balanced numbers of mild/severe TCRs) highlights the same findings as Fig 4B. We have added a sentence to the “Limitations of the study” paragraph on page 10 to address this point: “We find that a higher proportion of TCR sequences from mild cases converge with those from pre-pandemic individuals; however, it may be possible that this observation has arisen from higher numbers of TCRs from mild patients used as input for this convergence analysis.” We have now provided age information (please see R1); it is unlikely the differences seen have arisen due to age.

C4: The TCR avidity results are interesting, and the assay of T cell killing on virally-infected T cells provides a robust assessment of potential protective capacity. However, using this assay to derive firm conclusions on whether patients who recovered from mild disease maintain more high-avidity clones requires a higher number of individuals. For example, in Fig. 7, one of the individuals who recovered from mild disease maintained both high and low avidity clones similar to the proportions seen in the severe patients analyzed by scRNAseq (Fig. 6).

R4: We agree that any firm conclusion on whether patients who recovered from mild disease maintain more high avidity clones requires a much larger cohort – we have now emphasised this point in the “Limitations of this study” section of the manuscript on page 9/10: “Although our data supports that high frequency naive T cell precursors are likely to contribute to mild disease outcome, it is also possible as the consequence of high viral load and overstimulation caused by high functional avidity T cells (with higher proportion of precursor TCRs) leading to exhaustion and depletion during the acute virus infection, which merits further investigation including larger cohorts sizes.”

We are pleased that this reviewer thinks the assay of T cell killing on virally infected T cells provides a robust assessment of potential protective capacity. In the revised manuscript, we provided new additional data to evaluate the anti-viral efficacy of the T cell response against five VOC including the Delta variant (new Figure 7B, 7E and S5).

Reviewer #2:

C: Remarks to the Author:

Peng and co-workers performed an in-depth analysis of SARS-CoV-2 specific CD8+ T cells targeting the HLA-B*07:02-restricted NP105-113 epitope that has been described to be immunodominant by several groups. While this epitope-specific CD8+ T cell response has already been specifically studied in two previous papers published in IMMUNITY, the current manuscript clearly further extends the previous findings by adding important novel aspects. First, it links the strength of the response (but not presence of HLA-B*07:02 per se) to a mild course of infection. Second, they demonstrate that epitope-specific CD8+ T cells in patients with severe course display higher cytotoxicity and inhibitory receptor expression compared to patients with mild course. Third, TCRs from patients with mild course have higher similarity to TCRs from naïve precursors found in pre-pandemic samples and display a higher avidity. Fourth, the relevance of higher avidity TCRs is confirmed in natural processing as well as in vitro infection experiments. Finally, a proportionally narrowing of the TCR repertoire six months after infection comes along with maintenance of antiviral efficacy against prototype as well as variant (alpha, beta, gamma) virus. Thus, this study gives important insights into protective CD8+ T cell immunity in natural SARS-CoV-2 infection. There are, however, also some issues that need additional attention.

R: We appreciate the precise summary and highlighting of the importance of our paper.

Specific Comments:

C1: The authors state e.g. in the abstract "strong association of this response with mild disease". It is extremely important, however, to clearly state throughout the manuscript that the strength of the response, but not the presence of the response per se, is associated with mild disease. In this context, it is important to point out that Fig. 1b is extremely misleading/ incorrect. This panel refers to the 15 B*07:02+ patients who respond to the epitope. 9 of these (60%) had mild disease, 6 (40%) severe disease. This distribution is completely the same as the distribution in the overall cohort (30 mild cases; 22 severe cases). Thus, responding to the epitope per se is not associated with mild infection. The data displayed in Fig. 1B thus (1) should be displayed as a pie chart and (2) needs to be compared to the overall cohort.

R1: We apologise for the confusion this figure has caused and appreciate the suggestion of replacing with a pie chart.

We agree that the distribution of mild and severe in HLA-B*07:02 responders are similar to the overall cohort; however, the distribution is different from overall HLA-B*07:02 positive donors

consisting of 10 mild (9 responded to the epitope) and 9 severe disease (6 responded to the epitope). We have added new pie charts to clarify this information: an updated Figure 1B (responders in overall HLA-B*07:02 donor studied stratified by individuals recovered from mild and severe disease), and a new Supplementary Figure 1D (overall cohort and in HLA-B*07:02 positive individuals with mild and severe disease). The proportion of responders in mild cases (N=9/10, 90%) among HLA-B*07:02 individuals is greater than in severe disease (N=6/9, 67%), which further supports the association of this response with mild disease in addition to Figures 1A, C and D. We have now changed the text accordingly.

Figure 1B

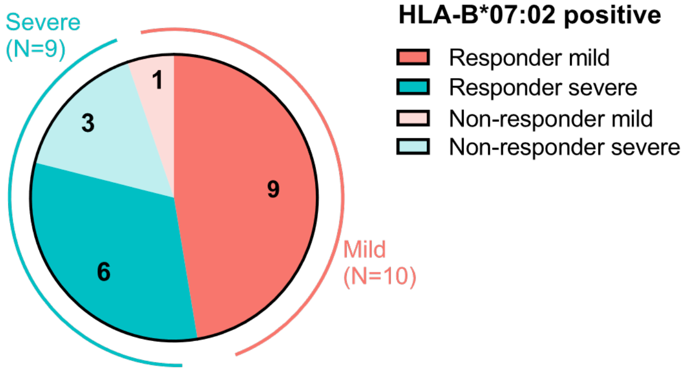

Legend, Figure 1: "**Frequency and magnitude of response to NP$_{105-113}$-B*07:02 epitope in COVID-19 patients**. (**A**) Frequency of convalescent COVID-19 patients (N=52 total patient cohort, N=19 HLA-B*07:02 positive patients only) with T cells responding to NP$_{105-113}$-B*07:02 epitope stimulation. (**B**) Frequency of HLA-B*07:02 positive responders (N=15) and non-responders (N=4) with mild or severe convalescent COVID-19 disease."

Supplementary Figure 1D

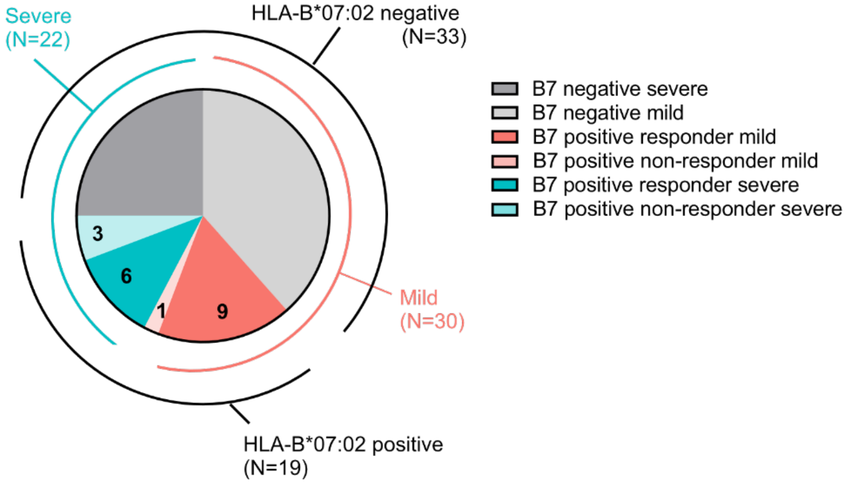

Legend, Supplementary Figure 1D: "(D) Breakdown of patient numbers and severity; HLA-B*07:02 negative (mild N=20 light grey, N=13 dark grey); HLA-B*07:02 positive responders (mild N=9 red, severe N=6 blue); HLA-B*07:02 positive non-responders (mild N=1 light red, severe N=3 light blue)."

Results, page 4: "We proceeded to carry out *ex vivo* IFN-γ ELISpot assays using HLA-B*07:02 positive convalescent samples 1-3 months post infection. 79% (15/19) of HLA-B*07:02 individuals showed responses to this epitope which accounted for 29% individuals of overall cohort (15/52) (Figure 1A), including 90% (9/10) of individuals recovered from mild and 67% (6/9) from severe disease (Figure 1B). This further confirms the dominance of this $NP_{105\text{-}113}$-B*07:02 T cell response in our cohort, in particular in individuals recovered from mild illness. In addition, individuals recovered from mild disease made significantly stronger responses to this epitope, compared to those who had recovered from severe disease (Figure 1C, *P*=0.04). We also observed that this $NP_{105\text{-}113}$-B*07:02-specific response is dominant in mild cases and makes up 60% of overall NP responses of each individual, whereas in severe cases, the proportion is substantially lower, with an average of 19.5% (Figure 1D, *P*=0.015). In addition, we did not find HLA-B*07:02 association with disease outcome in our study cohorts (Figure 1E, 77 acute and 52 convalescent patients). Our data highlights the association of the strength of this dominant epitope-induced T cell response with mild disease outcome and provides evidence that this link is epitope specific rather than a wider allelic association with HLA-B*07:02."

C2: The authors argue that higher cytotoxicity gene expression scores as well as increased inhibitory receptor expression in patients with severe infection argue for exposure to higher

antigen load, meaning that they are a consequence rather than a cause of severe infection. The same may be due to TCR similarity/divergence from naïve precursors as well as avidity, e.g. these may be a consequence rather than cause for mild versus severe disease. This limitation should be discussed more carefully.

R2: We appreciate and agree with this suggestion. A sentence has now been added to the "Limitations of the study" paragraph on page 9/10: "Although our data supports that high frequency naive T cell precursors are likely to contribute to mild disease outcome, it is also possible as the consequence of high viral load and overstimulation caused by high functional avidity T cells (with higher proportion of precursor TCRs) leading to exhaustion and depletion during the acute virus infection, which merits further investigation including larger cohorts sizes."

C3: 3. Fig. 7D: VOC alpha, beta, and gamma are studied here, but delta is missing. Since delta is the most relevant VOC at presence, it should also be analyzed here.

R3: We have now obtained the Delta variant from our collaborators and performed intracellular cytokine staining by co-culturing patient bulk T cell lines with autologous B cells infected by all VOCs. New figures 7B and S5 are now included in the revised manuscript, showing that T cell lines generated from six months after infection can respond very well to Victoria and Delta virus infected cells, as well as three other variants (Alpha, Beta, Gamma). We further demonstrate that bulk T cell lines could suppress Delta virus infected cells (new Figure 7E). We believe the new data provide additional evidence of the protective role of $NP_{105-113}$-specific T cells in secondary infection against different SARS-CoV-2 variants.

nature research

Figure 7B

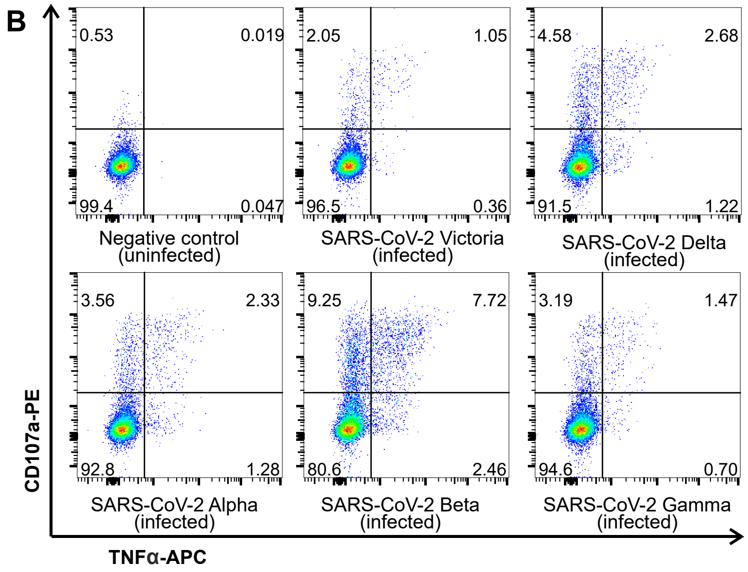

Legend, Figure 7B: "Representative ICS flow cytometry plots measuring TNF-α and CD107a expression on bulk NP105-113-specific T cell lines from C-COV19-046 incubated with SARS-CoV-2 Victoria, Alpha, Beta, Gamma or Delta variant infected B cell lines."

Figure 7E

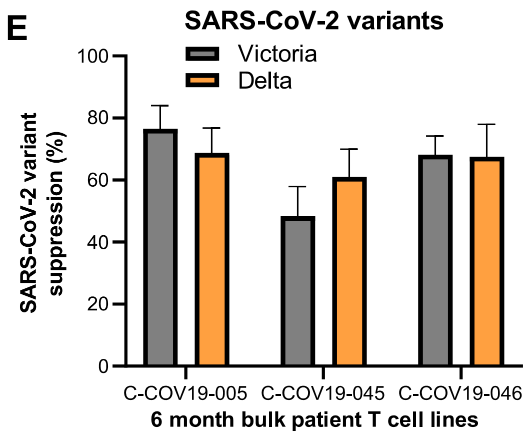

Legend, Figure 7E: “Anti-viral activity of NP105-113-specific bulk T cells from six months convalescence against SARS-CoV-2 variants of concern: Victoria strain (grey bars) and Delta variant (orange bars). Single representative experiment shown as means ± S.D.”

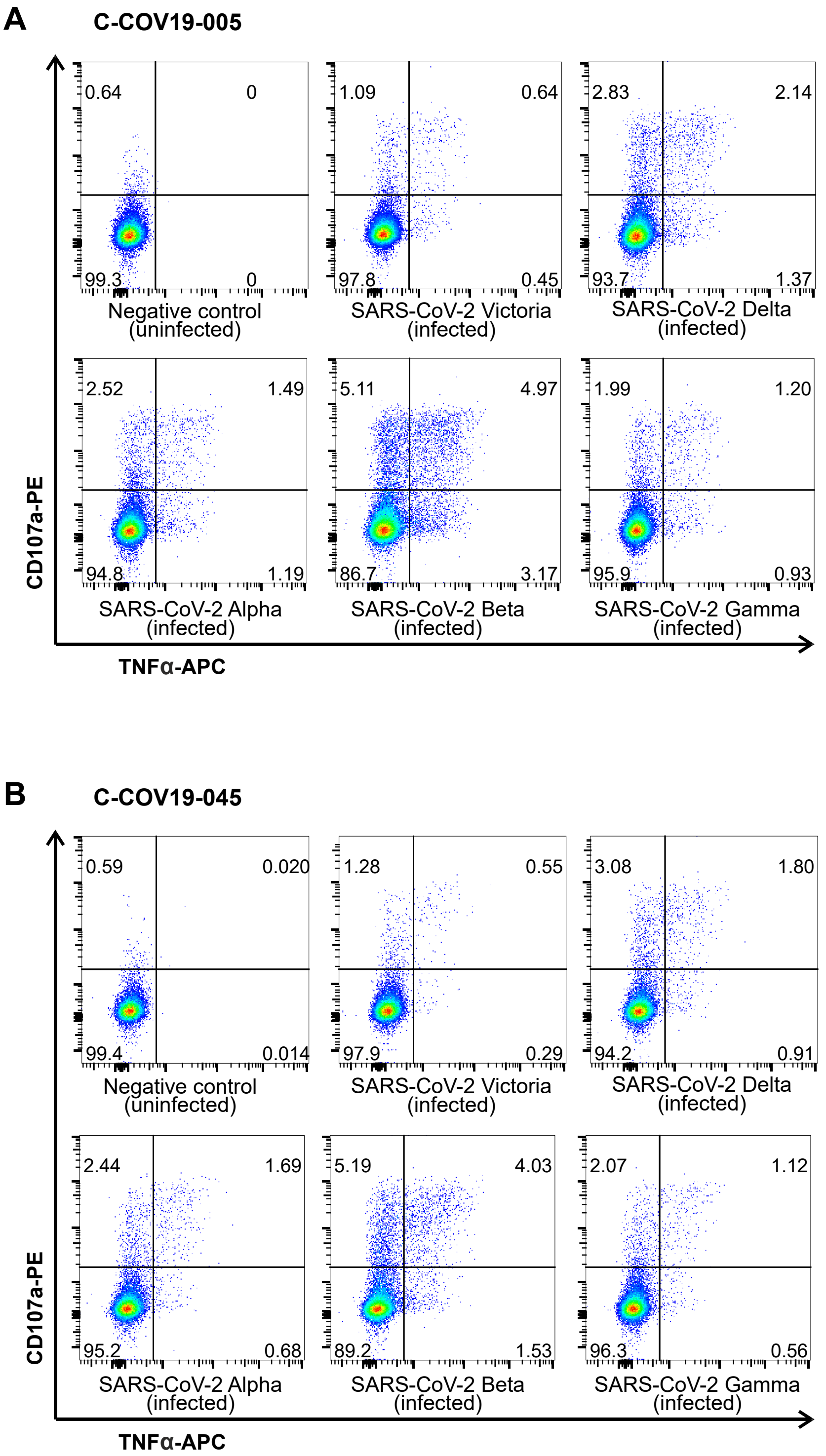

Legend, Supplementary Figure 5: “Representative ICS flow cytometry plots showing TNFα and

CD107a protein expression in patient-derived bulk T cell lines from six months convalescent samples after incubation with SARS-CoV-2 Victoria strain and VOCs (Alpha, Beta, Gamma and Delta) infected autologous B cell lines (A) patient C-COV19-005 and (B) C-COV19-045."

Results, page 8: "All three T cell lines showed increased MIP1β and CD107a protein expression after incubation with NP-expressing Vaccinia virus (Figure S4), increased TNF-α and CD107a expression after incubation with BCLs infected with SARS-CoV-2 virus (Victoria strain) and current variants of concerns (VOCs) including the Delta variant (Figure 7B, Figure S5). In addition, we found that these antigen-specific bulk cell lines are capable of suppressing SARS-CoV-2 replication (Figure 7C) and showed strong inhibition against VOCs, including the recently emerged Alpha, Beta, Gamma and Delta SARS-CoV-2 variants (Figure 7D-E)."

C4: Minor:

C4.1: HLA-B*07:02 is incorrectly spelled as HLA-B*0702 at multiple sites of the manuscript.

R4.1: This has been corrected to HLA-B*07:02

C4.2: 2. Line 164: "…by other studies (ref 7)..": Are there additional studies to ref 7?

R4.2: We have added the Lineburg *et al*. reference in addition to Nguyen *et al*. reference

C4.3: Line 179: "Both studies…": There are four references – please clarify.

R4.3: We have changed the wording to "These studies" instead of "Both studies"

C4.4: Lines 182-184: "Overall, our data supported the notion… (ref 7)": This sentence is unclear to me. Unexposed individuals have not been studied here, and ref 7 does not contain data by the authors of this study.

R4.4: We have moved this sentence to the end of this results section, page 6: "Cellular subtyping of these $CD8^+$ $NP_{105-113}$-B*07:02 T cells show a higher proportion of naïve T cells in one HLA-B*07:02 healthy individual compared to predominantly T effector memory subtypes in acute COVID-19 patients (N=17, Figure 4C). Overall, our data supports the report that T cells bearing TCRs specific to $NP_{105-113}$-HLA-B*07:02 in SARS-CoV-2 unexposed individuals are unlikely to have resulted from previous seasonal coronavirus infection [7]. This reinforces the finding that only $NP_{105-113}$-B*07:02 specific T cells from acute HLA-B*07:02 positive patients are exposed to antigen and undergo T cell differentiation, whereas $NP_{105-113}$-specific T cells in pre-

pandemic individuals are naïve precursors rather than memory cells from prior cross-reactive infection."

C4.5: Discussion, lines 312-313: "We found high similarity of TCR in both HLA-B*07:02 positive and HLA-B*07:02 negative COVID-19 recovered individuals…": What does this "similarity" refer to? The sentence is not clear to me.

R4.5: We have changed this sentence (Discussion, page 8) to "We found high similarity and convergence of TCRs in HLA-B*07:02 positive healthy and recovered individuals, with naive precursors identified in pre-pandemic samples supporting previous reports [7, 10]." to reflect the data shown in Fig 4A and 4B.

C4.6: Discussion, lines 359-362: "…high frequency naïve T cell precursors recognizing NP105-113-B*07:02 that are observed in pre-pandemic individuals are HLA-B*07:02 independent…": Where is this shown? Are they also present in HLA-B*07:02 negative individuals? This is not clear to me.

R4.6: Thank you for picking up this error, this sentence should have been deleted from an earlier draft. We have now replaced the sentence to: "firstly, we found strong association of $NP_{105-113}$-HLA-B*07:02 specific T cell response with mild disease"

We did find T cell precursors with naïve phenotypes in HLA-B*07:02 negative pre-pandemic individuals, these findings are not included in this manuscript as the number of TCRs detected are very low. We feel more data are needed to draw a firmer conclusion, and the data are not relevant to the conclusions of this paper.

Decision Letter, first revision:

Subject: Your manuscript, NI-A32469B

Message: Our ref: NI-A32469B

6th Oct 2021

Dear Dr. Dong,

Thank you for your patience as we've prepared the guidelines for final submission of your Nature Immunology manuscript, "An immunodominant NP105-113-B*07:02 cytotoxic T cell response controls viral replication and is associated with less severe COVID-19 disease" (NI-A32469B). Please carefully follow the step-by-step instructions provided in the attached file, and add a response in each row of the table to indicate the changes that you have made. Please also check and comment on any additional marked-up edits we

have proposed within the text. Ensuring that each point is addressed will help to ensure that your revised manuscript can be swiftly handed over to our production team.

We would like to start working on your revised paper, with all of the requested files and forms, as soon as possible (preferably within two weeks). Please get in contact with us if you anticipate delays.

When you upload your final materials, please include a point-by-point response to any remaining reviewer comments and please make sure to upload your checklist.

If you have not done so already, please alert us to any related manuscripts from your group that are under consideration or in press at other journals, or are being written up for submission to other journals (see: https://www.nature.com/nature-research/editorial-policies/plagiarism#policy-on-duplicate-publication for details).

In recognition of the time and expertise our reviewers provide to Nature Immunology's editorial process, we would like to formally acknowledge their contribution to the external peer review of your manuscript entitled "An immunodominant NP105-113-B*07:02 cytotoxic T cell response controls viral replication and is associated with less severe COVID-19 disease". For those reviewers who give their assent, we will be publishing their names alongside the published article.

Nature Immunology offers a Transparent Peer Review option for new original research manuscripts submitted after December 1st, 2019. As part of this initiative, we encourage our authors to support increased transparency into the peer review process by agreeing to have the reviewer comments, author rebuttal letters, and editorial decision letters published as a Supplementary item. When you submit your final files please clearly state in your cover letter whether or not you would like to participate in this initiative. Please note that failure to state your preference will result in delays in accepting your manuscript for publication.

Cover suggestions

As you prepare your final files we encourage you to consider whether you have any images or illustrations that may be appropriate for use on the cover of Nature Immunology.

Covers should be both aesthetically appealing and scientifically relevant, and should be supplied at the best quality available. Due to the prominence of these images, we do not generally select images featuring faces, children, text, graphs, schematic drawings, or collages on our covers.

We accept TIFF, JPEG, PNG or PSD file formats (a layered PSD file would be ideal), and the image should be at least 300ppi resolution (preferably 600-1200 ppi), in CMYK colour mode.

If your image is selected, we may also use it on the journal website as a banner image, and may need to make artistic alterations to fit our journal style.

Please submit your suggestions, clearly labeled, along with your final files. We'll be in touch if more information is needed.

Nature Immunology has now transitioned to a unified Rights Collection system which will allow our Author Services team to quickly and easily collect the rights and permissions required to publish your work. Approximately 10 days after your paper is formally accepted, you will receive an email in providing you with a link to complete the grant of rights. If your paper is eligible for Open Access, our Author Services team will also be in touch regarding any additional information that may be required to arrange payment for your article.

You will not receive your proofs until the publishing agreement has been received through our system.

Please note that <i>Nature Immunology</i> is a Transformative Journal (TJ). Authors may publish their research with us through the traditional subscription access route or make their paper immediately open access through payment of an article-processing charge (APC). Authors will not be required to make a final decision about access to their article until it has been accepted. Find out more about Transformative Journals.

If you have any questions about costs, Open Access requirements, or our legal forms, please contact ASJournals@springernature.com.

<B>Authors may need to take specific actions to achieve compliance with funder and institutional open access mandates. For submissions from January 2021, if your research is supported by a funder that requires immediate open access (e.g. according to Plan S principles) then you should select the gold OA route, and we will direct you to the compliant route where possible. For authors selecting the subscription publication route our standard licensing terms will need to be accepted, including our self-archiving policies. Those standard licensing terms will supersede any other terms that the author or any third party may assert apply to any version of the manuscript.

Please use the following link for uploading these materials: [REDACTED]

If you have any further questions, please feel free to contact me.

Best regards,

Elle Morris
Senior Editorial Assistant
Nature Immunology
Phone: 212 726 9207
Fax: 212 696 9752
E-mail: immunology@us.nature.com

On behalf of

Zoltan Fehervari, Ph.D.
Senior Editor
Nature Immunology

The Macmillan Building
4 Crinan Street
Tel: 212-726-9207
Fax: 212-696-9752
z.fehervari@nature.com

Reviewer #1:
Remarks to the Author:
The authors have addressed the major concerns and added new results on T cell reactivity to SARS-CoV-2 variants of concern.

Reviewer #2:
Remarks to the Author:
The authors have carefully addressed all my comments, also by performing additional experiments and by showing additional data. I have no further comments.

Final Decision Letter:

Subject: Decision on Nature Immunology submission NI-A32469C
Message: In reply please quote: NI-A32469C

Dear Dr. Dong,

I am delighted to accept your manuscript entitled "An immunodominant NP105-113-B*07:02 cytotoxic T cell response controls viral replication and is associated with less severe COVID-19 disease" for publication in an upcoming issue of Nature Immunology.

The manuscript will now be copy-edited and prepared for the printer. Please check your calendar: if you will be unavailable to check the galley for some portion of the next month, we need the contact information of whom will be making corrections in your stead. When you receive your galleys, please examine them carefully to ensure that we have not inadvertently altered the sense of your text.

Acceptance is conditional on the data in the manuscript not being published elsewhere, or announced in the print or electronic media, until the embargo/publication date. These restrictions are not intended to deter you from presenting your data at academic meetings and conferences, but any enquiries from the media about papers not yet scheduled for publication should be referred to us.

Please note that <i>Nature Immunology</i> is a Transformative Journal (TJ). Authors

may publish their research with us through the traditional subscription access route or make their paper immediately open access through payment of an article-processing charge (APC). Authors will not be required to make a final decision about access to their article until it has been accepted. Find out more about Transformative Journals.

<B>Authors may need to take specific actions to achieve compliance with funder and institutional open access mandates. For submissions from January 2021, if your research is supported by a funder that requires immediate open access (e.g. according to Plan S principles) then you should select the gold OA route, and we will direct you to the compliant route where possible. For authors selecting the subscription publication route our standard licensing terms will need to be accepted, including our self-archiving policies. Those standard licensing terms will supersede any other terms that the author or any third party may assert apply to any version of the manuscript.

In approximately 10 business days you will receive an email with a link to choose the appropriate publishing options for your paper and our Author Services team will be in touch regarding any additional information that may be required.

You will not receive your proofs until the publishing agreement has been received through our system.

If you have any questions about our publishing options, costs, Open Access requirements, or our legal forms, please contact ASJournals@springernature.com

Once your manuscript is typeset and you have completed the appropriate grant of rights, you will receive a link to your electronic proof via email with a request to make any corrections within 48 hours. If, when you receive your proof, you cannot meet this deadline, please inform us at rjsproduction@springernature.com immediately. Once your paper has been scheduled for online publication, the Nature press office will be in touch to confirm the details.

Your paper will be published online soon after we receive your corrections and will appear in print in the next available issue. Content is published online weekly on Mondays and Thursdays, and the embargo is set at 16:00 London time (GMT)/11:00 am US Eastern time (EST) on the day of publication. Now is the time to inform your Public Relations or Press Office about your paper, as they might be interested in promoting its publication. This will allow them time to prepare an accurate and satisfactory press release. Include your manuscript tracking number (NI-A32469C) and the name of the journal, which they will need when they contact our office.

About one week before your paper is published online, we shall be distributing a press release to news organizations worldwide, which may very well include details of your work. We are happy for your institution or funding agency to prepare its own press release, but it must mention the embargo date and Nature Immunology. Our Press Office will contact you closer to the time of publication, but if you or your Press Office have any

enquiries in the meantime, please contact press@nature.com.

Also, if you have any spectacular or outstanding figures or graphics associated with your manuscript - though not necessarily included with your submission - we'd be delighted to consider them as candidates for our cover. Simply send an electronic version (accompanied by a hard copy) to us with a possible cover caption enclosed.

To assist our authors in disseminating their research to the broader community, our SharedIt initiative provides you with a unique shareable link that will allow anyone (with or without a subscription) to read the published article. Recipients of the link with a subscription will also be able to download and print the PDF.

As soon as your article is published, you will receive an automated email with your shareable link.

You can now use a single sign-on for all your accounts, view the status of all your manuscript submissions and reviews, access usage statistics for your published articles and download a record of your refereeing activity for the Nature journals.

If you have not already done so, we strongly recommend that you upload the step-by-step protocols used in this manuscript to the Protocol Exchange. Protocol Exchange is an open online resource that allows researchers to share their detailed experimental know-how. All uploaded protocols are made freely available, assigned DOIs for ease of citation and fully searchable through nature.com. Protocols can be linked to any publications in which they are used and will be linked to from your article. You can also establish a dedicated page to collect all your lab Protocols. By uploading your Protocols to Protocol Exchange, you are enabling researchers to more readily reproduce or adapt the methodology you use, as well as increasing the visibility of your protocols and papers. Upload your Protocols at www.nature.com/protocolexchange/. Further information can be found at www.nature.com/protocolexchange/about .

Please note that we encourage the authors to self-archive their manuscript (the accepted version before copy editing) in their institutional repository, and in their funders' archives, six months after publication. Nature Research recognizes the efforts of funding bodies to increase access of the research they fund, and strongly encourages authors to participate in such efforts. For information about our editorial policy, including license agreement and author copyright, please visit www.nature.com/ni/about/ed_policies/index.html

An online order form for reprints of your paper is available at https://www.nature.com/reprints/author-reprints.html. Please let your coauthors and your institutions' public affairs office know that they are also welcome to order reprints by this method.

Sincerely,

Zoltan Fehervari, Ph.D.
Senior Editor
Nature Immunology

natureresearch

The Macmillan Building
4 Crinan Street
Tel: 212-726-9207
Fax: 212-696-9752
z.fehervari@nature.com
